# Dietary, socioeconomic, and maize handling practices associated with aflatoxin and fumonisin exposure among women tortilla makers in 5 departments in Guatemala

**DOI:** 10.1371/journal.pgph.0001623

**Published:** 2024-02-07

**Authors:** Ariel V. Garsow, Olga R. Torres, Jorge A. Matute, Danielle M. Voss, Gonzalo Miyagusuku-Cruzado, M. Monica Giusti, Barbara B. Kowalcyk

**Affiliations:** 1 Department of Food Science and Technology, The Ohio State University, Columbus, Ohio, United States of America; 2 Center for Foodborne Illness Research and Prevention, The Ohio State University, Columbus, Ohio, United States of America; 3 Laboratorio Diagnóstico Molecular, Guatemala City, Guatemala; 4 Centro De Investigación en Nutrición y Salud, Guatemala City, Guatemala; 5 Translational Data Analytics Institute, The Ohio State University, Columbus, Ohio, United States of America; Sciensano, BELGIUM

## Abstract

Previous research has demonstrated human exposure to mycotoxins among Guatemalans, with high levels of mycotoxins being found in blood and urine samples as well as in maize for human consumption. Mishandling of crops such as maize during pre- and post-harvest has been associated with mycotoxin contamination. The overarching goal of this study was to identify risk factors for aflatoxin and fumonisin exposure in Guatemala. A cross-sectional survey of 141 women tortilla makers was conducted in the departments of Guatemala, Sololá, Suchitepéquez, Izabal, and Zacapa in February 2022. Maize and tortilla samples were collected and analyzed for aflatoxin B_1_ (AFB_1_) and fumonisin B_1_, B_2_, and B_3_ contamination (FB_1_, FB_2_, FB_3_). Urine samples were collected and analyzed for urinary FB_1_ (uFB_1_) contamination. A questionnaire was administered to collect data on sociodemographic characteristics, dietary intake of maize-based foods the week prior to the study, and maize handling practices. Descriptive statistics were used to describe common maize handling practices. A univariable analysis was conducted to identify predictors of low/high AFB_1_, total fumonisins, and uFB_1_. Multivariable logistic regression was used to calculate adjusted odds ratios (ORs) and 95% confidence intervals (CIs). During tortilla processing, a reduction in the AFB_1_ and total fumonisin levels was observed. The presence of AFB_1_ in maize was associated with department and mean total fumonisin level in maize (OR: 1.705, 95% CI: 1.113–2.613). The department where the tortilleria was located was significantly associated with the presence of fumonisins in tortillas. Increased consumption of Tortrix was significantly associated with the presence of FB_1_ in urine (OR: 1.652, 95% CI: 1.072–2.546). Results of this study can be used in the development and implementation of supply chain management practices that mitigate mycotoxin production, reduce food waste and economic loss, and promote food security.

## Introduction

Mycotoxins, such as aflatoxins and fumonisins, are a serious food security and public health threat globally. Consumption of food contaminated with high levels of mycotoxins, such as aflatoxins and fumonisins, has been associated with serious acute and long-term health outcomes such as acute hepatitis, hepatocellular carcinoma (liver cancer), and stunting [1–6]. Mycotoxin contamination is a significant problem in low- and middle-income countries (LMICs), which frequently have hot, humid climates that promote mycotoxin production. In LMICs, like Guatemala, aflatoxin and fumonisin exposure can be quite high due to mishandling of maize during storage and processing, insufficient regulatory standards and analytical capacity for monitoring contamination, and lack of diversity in diets [6–8]. Maize handling practices, such as nixtamalization, can potentially reduce mycotoxin contamination in maize [9–14].

Aflatoxins are secondary metabolites primarily produced by the fungi *Aspergillus flavus* in crops such as maize. In the Americas, the most common aflatoxin found on maize is aflatoxin B_1_ (AFB_1_), a Group 1 carcinogen [3,5,15,16]. Given its DNA reactivity, any level of exposure could increase the risk of tumor formation, and thus, aflatoxin contamination levels should be as low as reasonably achievable [17,18].

Fumonisins are secondary metabolites primarily produced by *Fusarium verticillioides* in crops such as maize. The most common forms of fumonisins found on maize are Fumonisin B_1_, B_2_, and B_3_ (FB_1_, FB_1_, and FB_3_) [19,20]. FB_1_ is a Group 2B carcinogen [18]. Fumonisin exposure has been associated with stunting and neural tube defects [1,2,6,21]. The provisional maximum tolerable daily intake of fumonisins set by the World Health Organization is 2 μg/kg body weight per day [18,22,23].

Aflatoxins and fumonisins have high thermal stability making heat treatment a non-viable intervention for mitigating risk of exposure [24,25]. Consequently, mitigation practices focus on prevention and decontamination. Prevention of mycotoxin contamination includes the use of agricultural products, limiting insect damage, and ensuring proper drying and storage conditions, which have been demonstrated to limit fungal growth and subsequent toxin production [26,27]. Decontamination of foods can occur through processing methods such as nixtamalization, a traditional process for making masa for corn-based products that involves soaking maize in an alkaline solution to remove the exterior coat of the maize kernel, open the lactone ring of aflatoxins, and hydrolyze fumonisins through the removal of carboxylic acid [9–13,28]. There is conflicting scientific evidence regarding the effects of nixtamalization on aflatoxin and fumonisins which depends on the nixtamalization method used [29,30], requiring additional research.

Mycotoxins have been identified as a hazard to women and children’s health in Guatemala. The mean prevalence of stunting (a condition that interferes with brain development) in Guatemala is estimated to be 47% for children under 5 years of age [31]. Exposure to mycotoxins has been associated with stunting in other countries [2,21,32–34]. In the most recent maize survey conducted in Guatemala in 2012, co-exposure between aflatoxins and fumonisins in maize samples was common [8]. The mean aflatoxin and fumonisin concentrations ranged from 0.4–262 μg/kg and 0.6–4.7 μg/Kg, respectively. Additionally, a preliminary analysis of urine samples from this study found that 15% were positive for aflatoxin M_1_, 48% were positive for uFB_1_, and 11.0% were positive for both aflatoxin M_1_ and uFB_1_ [8].

The Guatemalan government has recognized that consumption of foods contaminated with mycotoxins has been associated with negative health outcomes. The Guatemalan Commission of Norms (COGUANOR) has set 20 parts per billion (ppb) as the action level for aflatoxins in maize [35]. There is currently no action level for fumonisins although the FAO/WHO Expert Committee on Food Additives has set a provisional maximum tolerable daily intake (JECFA PMTDI) for total fumonisins (FB_1_, FB_2_, and FB_3_) of 2 μg/kg body weight (bw)/day [18].

In Guatemala, where maize is a staple crop and the most frequently consumed food, exposure to aflatoxins and fumonisins is high. Previous studies have found high levels of aflatoxins in maize throughout Guatemala as well as the presence of the AFM_1_ biomarker in human urine samples [8,23,36–39]. Different post-harvest maize handling practices have been found in Guatemala [40,41], which may also contribute to high exposure. The objective of this study was to estimate routes of aflatoxin and fumonisin exposure in women tortilla makers in Guatemala to inform future research around control of aflatoxin and fumonisin contamination in the food supply chain. The main hypothesis of this research is that certain maize storage and processing practices impact aflatoxin and fumonisin levels in corn and tortillas and subsequent exposure to fumonisins in Guatemala.

## Methods

### Ethics statement

The study had ethical approval from the Institutional Review Boards (IRB) in Guatemala (Hospital Roosevelt; CODEIHR. No. 35) and in the United States (Ohio State University; Study Number: 2021H0234). The field workers were trained according to the standards of both IRBs and the researchers met all the requirements of both committees. Written informed consent or a fingerprint demonstrating consent was obtained by the data collectors from all the prospective participants. Study participants were paid the market price for the tortilla and corn samples collected and received a bar of soap.

### Study design

Women tortilla makers from five departments (first-level geopolitical administrative areas in Guatemala) of the Republic of Guatemala (the departments of Sololá, Suchitepéquez, Izabal Zacapa, and Guatemala) were recruited in February 2022 to participate in this cross-sectional study. These departments were chosen to represent a range of aflatoxin and fumonisin exposure levels in maize as estimated by the last maize survey conducted in the country [42].

Thirty women tortilla makers were recruited from each department with representation from each municipality (second-level geopolitical administrative areas in Guatemala) except when it was deemed unsafe for the study team. Eligible study participants were women tortilla makers over 18 years of age who consumed tortillas from the tortilleria (tortilla shop) in which she worked. To identify eligible participants, the study team visited the main market town in each municipality and developed a list of tortillerias. To develop the list of tortillerias, the study team started from the main square or road of the town and systematically walked or drove down every street in the town writing down the names and locations of all of the tortillerias in that town. If the town border was unclear, the study team received clarification from a resident of that town. The study team then used a list of random numbers to randomly select a tortilleria. The tortilleria was visited to determine if any of the workers were eligible and willing to participate in the study. If none of the workers were eligible or willing to participate, the study team randomly selected another tortilleria from the list and continued to do so until an eligible participant was identified and consented.

### Questionnaire administration

Questionnaires were used to collect data on sociodemographic characteristics, maize handling practices, and dietary consumption patterns (S1 Material, S2 Material). Given the low literacy rates among Guatemalan women, questionnaires were completed via face-to-face interviews conducted by trained field workers who speak Spanish or Spanish-Kíche (Mayan dialect). When a participant spoke another dialect, the interview was conducted with the help of local translators.

Data on sociodemographic characteristics and maize handling practices were collected. Sociodemographic data included marital status, education level (above or below high school), age, and language spoken. The number of people working in the tortilla shop as well as the material of the walls, floor, and ceiling was also recorded. Participants were also asked about their maize growing, purchasing, and storage practices including if they grow and purchase maize; how often they purchase maize; where they purchase maize; where the maize they purchase is harvested; and where they store the maize. Additionally, participants were asked about the quality of the maize that they purchased. Data collectors also observed the conditions of the maize storage as well as if the maize was whole or broken or had contaminants or foreign materials in it. Participants were asked about their tortilla preparation practices including: what color of maize they used to make masa; what do they do with maize that is contaminated with fungus or damaged by birds or insects; how do they prepare masa including the nixtamalization process used [what ingredients they use to make masa and the respective amounts of each, what type of water source they have, how long maize is boiled, and what type of comal (cooking surface) they use to make tortillas]; how many times they make tortillas per day; how many tortillas they make in a day; what do they do with leftover water used for nixtamalization and washing; and what do they do with the leftover masa.

In previous studies, maize-based foods were found to comprise the majority of the diet of women in Guatemala [8,22,23,42,43]. Therefore, in this study, women were also asked about their consumption of 19 locally produced (not highly processed), highly processed (most likely do not contain aflatoxins or fumonisins) or micronutrient fortified maize-based foods (Table 1) in the previous week using a food frequency questionnaire (FFQ) used in similar studies [8,22,23,42,43]. The amount consumed was estimated for each food item using known average weights of grams ingested (e.g.: one tamal, one tortilla, one bag of Tortrix, one cup, one tablespoon, etc.) [44].

**Table 1:** Food groups and serving size (g) for associated food items [44].

Group Name	Food Items Included in Group	Serving Size (g)
Locally produced maize-based foods	Boiled corn on the cob	115.00
	Chuchitos	75.00
	Corn atol (sweet beverage)	40.00
	Maize coffee	20.00
	Masa beverage	40.00
	Nachos	60.00
	Pinol	28.75
	Polenta (corn flour)	28.75
	Tacos	35.00
	Tamales	200.00
	Tamalitos	55.00
	Tayuyos	55.00
	Tortillas	40.0
	Tostadas	25.00
	Shepes	65.00
Highly processed maize-based foods^1^	Atol de maicena (starch atole)	40.00
	Corn flakes	45.00
	Tortrix	40.00
Micronutrient fortified maize-based foods^1^	Incaparina	36.00

^1^Processed so that fumonisin contamination is unlikely (purified starch, extrusion cooking, etc).

### Maize and tortilla sample collection and analysis

Maize and tortilla samples were collected to estimate the effects of processing on AFB_1_ and total fumonisin concentration (FB_1_, FB_2_, and FB_3_). Tortillerias that did not have maize available for purchase were asked where they purchased their maize and, when possible, the study team purchased maize from that maize seller.

Maize (approximately 500 g) and tortillas samples (2 tortillas) were placed into a brown paper bag and labelled. Tortillas were dried to a constant moisture content using a toaster (Black and Decker slide toaster, Beachwood, OH. USA) within 8 hours of sample collection to prevent further mold and fungal growth. Maize and dried tortillas were transported to a central laboratory and stored in a dry area.

Two 50 g sub-samples were taken and analyzed as biological replicates. The moisture content of maize samples was measured using a moisture content reader (M3G portable moisture content reader, Dickey-John Corporation, Auburn, IL, USA). AFB_1_ and total fumonisin concentration (FB1, FB_2_, and FB_3_) were measured using an enzyme-linked immunosorbent assay (AgraQuant Fumonisin 0.25/5.0 and AgraQuant Aflatoxin B_1_ ELISA kits, Romer Labs, Newark, DE, USA). The average of these values was used for further analysis.

Briefly, tortilla and maize samples were ground using a standard method. AFB_1_ and total fumonisins were extracted using a 70% methanol solution and filtered. The AFB_1_ or Fumonisin conjugate solution was mixed with the filtered extract, washed, and the substrate was added. A spectrophotometer was used to measure absorbance at 450 nm (differential filter 630 nm) and estimate AFB_1_ and total fumonisin concentration (FB_1_, FB_2_, and FB_3_) in the samples (Awareness, Palm City, Florida, USA). A standard curve using 0, 2, 5, 20, and 50 ppb AFB_1_ standard and 0, 0.25, 1.0, 2.5, and 5.0 ppm fumonisin standards was run with each test.

#### Urine sample collection and analysis

Individual exposure to FB_1_ was estimated using uFB_1_ (ng/mL) levels. Participants were asked to provide a urine sample following completion of the questionnaire. The study participant was instructed on how to collect the urine sample and given a sterile collection cup to provide the sample. Urine samples were analyzed following published protocols [22,42,45] with modifications. Briefly, urine samples (9 mL) were collected by the research staff and delivered to Laboratorio Diagnóstico Molecular in Guatemala City. Urine samples were adjusted to 10% acetonitrile using acetonitrile containing 1% formic acid. A total of 40 ng of U-[^13^C_34_]-FB_1_ (Romer Labs, Newark, DE, USA) was added to each urine sample as an internal standard to allow for quantification of uFB_1_ levels in samples after extraction. Fumonisins were isolated on C_18_ solid phase extraction (SPE) 360 mg sorbent cartridges (Sep-Pak R Classic C18 cartridges, Waters Corporation, Milford MA, USA). The loaded solid phase extraction cartridges were shipped to and eluted at The Ohio State University in Columbus, Ohio as described in Riley et al. [45] using 2 mL of 68.5% acetonitrile:30.5% water: 1% formic acid. Eluates were centrifuged using 0.2μm nylon micro-centrifugal filters, and the centrifuged portion was mixed with 1% formic acid in H_2_O to reach a solvent composition equivalent to the starting mobile phase gradient.

Reverse phase HPLC analysis was conducted using a Shimadzu, Nexera-i LC2040 3D UHPLC system (Shimadzu, Columbia, MD, USA). Chromatographic separation was accomplished using an Restek Raptor ARC-18 column (2.7 μm, 150 x 3.0 mm; Restek, Bellefonte, PA, USA) with a Raptor ARC-18 column guard (Restek, Bellefonte, PA, USA), under a binary gradient consisting of A: 97% water, 2% acetonitrile, 1% formic acid and B: 97% acetonitrile, 2% water, 1% formic acid. The following solvent gradient and flow rate was used for separation: 30–80% B from 0–7 min at 0.2 mL/min flow rate, 80–100% B from 7–8 min at 0.35 mL/min flow rate, 100% B from 8–11 min at 0.35 mL/min flow rate, followed by 5 minutes of column equilibration. The column effluent directly flowed to a Shimadzu LC8040 triple quadrupole mass spectrometer for detection of FB_1_ (Shimadzu, Columbia, MD, USA), run under 2.0 L/min nebulizing gas, 5 L/min drying gas flow, 160°C desolvation line temperature, and 200°C heat block temperature. Analytes were monitored under positive ionization with selective ion monitoring for 722.30 (for FB_1_) and 756.50 (for ^13^CFB_1_) and MRM fragmentation of 722.3 to 352.4 and 722.3 to 334.20 under -43 eV collision energy for FB_1_ and 756.5 to 374.2 and 756.5 to 356.1 under -45 eV collision energy for ^13^CFB_1_ determined based on preliminary work under the present experimental conditions. Each urine sample was injected once for uHPLC-MS/MS analysis. Throughout analysis, 6 samples were randomly selected to be injected a second time to help monitor system consistency. A standard curve of FB_1_ was prepared for each of the 4 sample shipments. Briefly, starting from a stock concentration of FB_1_ (Sigma Aldrich, St. Louis, MO, USA) dissolved and stored frozen at 50,000 pg/μL in 50% acetonitrile, 49% water, and 1% HCl, serial dilutions were performed to reach at least 5 concentrations between 0.5 to 60 pg/μL in the starting mobile phase solution FB_1_ standards were injected in duplicates with the order randomized throughout the sample run for each shipment.

Peaks were manually integrated based on retention time, comparison to standards, and presence of MRM, if applicable, and signal to noise ratio (S/N) was calculated by Shimadzu Lab Solutions software (version 5.80). Detection of FB_1_ was determined based on either a S/N ratio above 3.3 and/or an MRM signal. The limit of detection for uFB_1_ was ~1.15 ng/mL urine based on the determined S/N ratio for the FB_1_ standards. Urine samples were only analyzed for FB_1_ levels.

### Statistical analysis

Descriptive statistics were calculated for demographic, dietary, and maize-handling variables overall, by department, and by presence/absence of aflatoxins or fumonisins. Since distributions were right-skewed, nonparametric tests were used to assess differences between department and positivity aflatoxins or fumonisins. Distributions of categorical variables (department, language, education, marital status, age group, characteristics of tortilleria, type of maize storage, maize quality, frequency of purchasing maize, ingredients and equipment used to make tortillas, and tortilla making practices) were evaluated using chi-square tests. Differences in means of continuous variables (total fumonisin level in tortillas and maize, AFB_1_ level in maize and tortillas, total grams and servings of maize-based foods consumed, total grams and servings by food type, amount of maize stored) were evaluated using a Kruskal-Wallis test.

**Fig 1:**
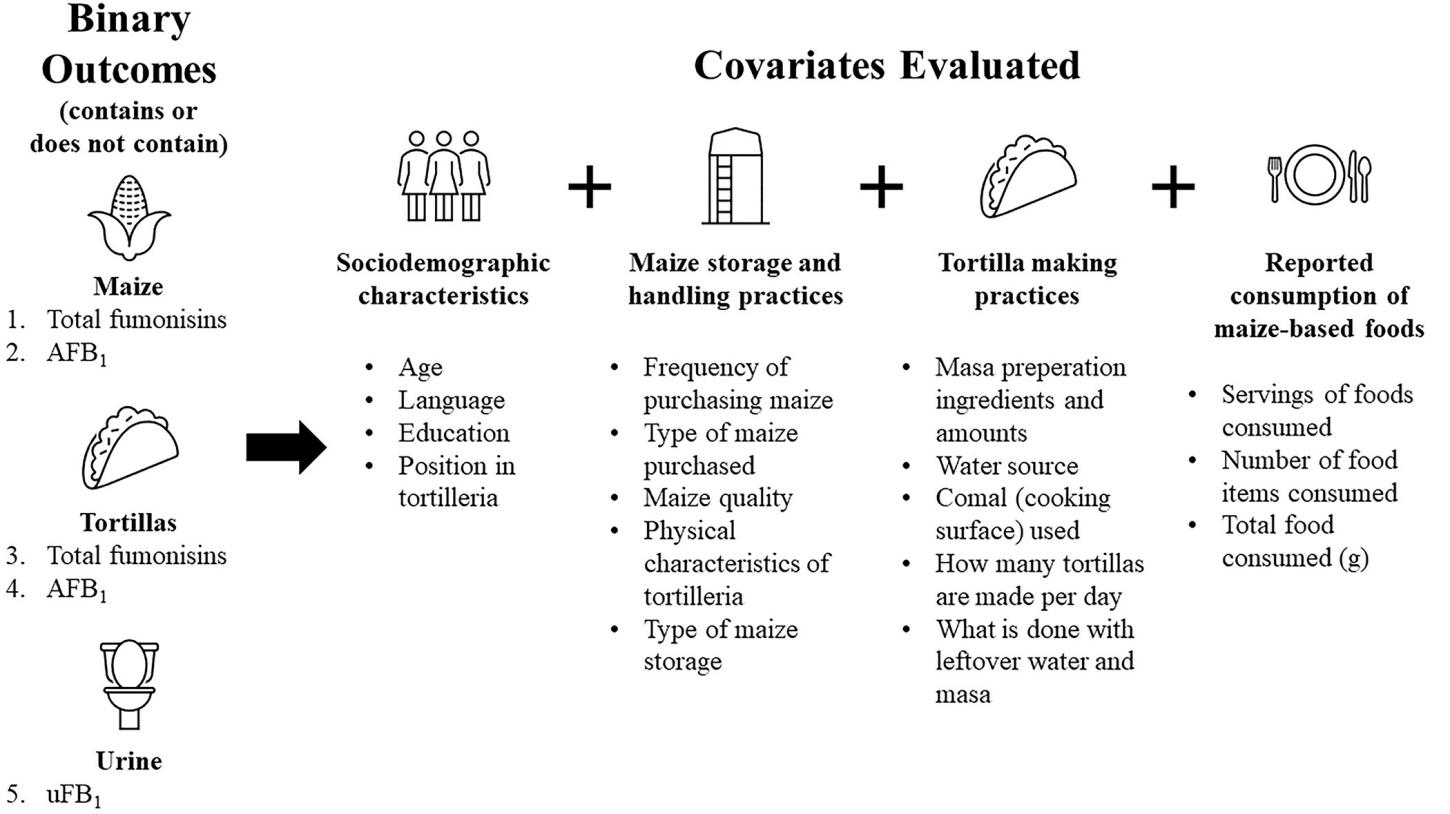
Logistic regression model outcomes and covariates evaluated. (Binary outcomes for total fumonisins and AFB_1_ were considered as presence or absence).

Univariable logistic regression was used to assess the relationship between the five outcomes (aflatoxin and fumonisin presence or absence in maize; aflatoxin and fumonisin presence or absence in tortillas; FB_1_ exposure group) and sociodemographic characteristics; maize consumption (total food consumed in grams, number of maize-based food items consumed, amount of maize-based food consumed in grams, number of food types consumed); maize storage and handling practices; and tortilla making practices (Fig 1). Odds ratios (ORs) and 95% confidence intervals (CIs) for food consumption variables reflect the change in odds of total fumonisin presence or absence in maize or tortillas, AFB_1_ presence or absence in maize or tortillas, or FB_1_ exposure with a one serving increase in food intake, or a one unit change in processing variables. Variables that had less than ten observations per category were not included in the univariable or multivariable analysis.

Multivariable logistic regression was used to calculate adjusted ORs and 95% CIs for comparing presence or absence of the five outcomes of total fumonisins and AFB_1_ in maize and tortillas, and uFB_1_. Variables significant at α = 0.20 in the univariable analysis were included in the multivariable analysis. The multivariable models were fit using backwards selection (Fig 1, Eq 1). All possible interactions between main effects were included.

**Eq 1** Example logistic regression model.


$$log\frac{Ρ}{1-Ρ}=\beta_{0}+\beta_{1\;}(Sociodeomographics)+\beta_{2}(Maize\;storage\;and\;handling)+\beta_{3}(Tortilla\;making)+\beta_{4}(Consumption)$$

Sensitivity analyses were conducted to evaluate the impact of imputing missing food consumption data. Imputing missing food consumption data as zero and the median amount consumed produced similar results (not presented).

With a sample size of 150 tortillerias (5 departments with 30 tortillerias each), this study had a 80% power to detect total aflatoxin contamination levels in maize samples greater than 20 ppb, assuming a standard deviation of 7.74 ppb [8,41], and 80% power to detect total aflatoxin contamination levels in tortillas greater than 2 μg/kg at a significance level of 0.05, assuming a standard deviation of 2.3 μg/kg [9,11,12,46].

Data were recorded by double entry and validation using Epi-Info 6.04d, and all statistical analyses were performed using SAS v.9.4 for Windows (SAS Institute Inc., USA). Statistical significance was defined to be p-value ≤ 0.5 and marginal significance was defined to be 0.5 < p-value ≤ 0.10. Ethical approval was obtained from the national IRB in Guatemala at Hospital Roosevelt and through the IRB at The Ohio State University.

## Results

A total of 150 women tortilla makers were recruited to participate in this study. Of these, 141 met the inclusion criteria and participated in the study. The number of study participants was similar in each department with 29 from the Guatemala department, 28 from Sololá, 30 from Suchitepéquez, 26 from Izabal, and 28 from Zacapa.

Most participants were the owners of the tortilleria (90.21%); spoke Spanish (89.51%); were over 35 years of age (60.14%) and had less than a high school education (76.92%) (Table 2).

**Table 2:** Sociodemographic characteristics of participants.

Characteristic		Overall (N = 141)	
		N	(%)
Primary language	Spanish	127	(90.07)
	Mayan	14	(9.92)
Age group	18–25 years	13	(9.22)
	25–29 years	25	(17.73)
	30–34 years	19	(13.48)
	35 years and above	84	(59.57)
Education	Less than high school	108	(95.61)
	High school and above	32	(22.38)
	Unknown	1	(0.01)
Position in tortilleria	Owner	127	(90.71)
	Family member	4	(2.86)
	Employee	9	(6.43)

On average, participants consumed 4.55 (SD: 2.31) types of maize-based foods comprising of 143 servings (SD: 2.31) or 3,470.53 g (SD: 2,050.21 g) of maize-based foods the week prior to the study. The most consumed maize-based food was tortillas (Table 3).

**Table 3:** Maize-based foods consumed in the week prior to study enrollment (mean percentage; other category includes foods with <1.00% consumption).

Maize-based Food	Percentage of Overall Diet (Mean)
Tortillas	82.97
Tamales	3.26
Incaparina	1.91
Chuchitos	1.54
Boiled sweet corn	1.18
Masa beverage	1.18
Tostadas	1.18
Tamalitos	1.15
Corn flakes	1.06
Other	4.56

Results are summarized below by step in maize-processing: maize contamination and maize-handling practices, tortilla contamination and tortilla making practices, and exposure. Sensitivity analyses for missing data did not produce significantly different results (not presented), so presented results use unimputed data. All possible interactions between main effects were tested but none were significant and, thus, were excluded from the final model.

### Maize contamination and maize-handling practices

Of the 123 maize samples collected, AFB_1_ was detected in 27.64% of samples, total fumonisins were detected in 77.23% of samples, and both fumonisin and aflatoxins were detected in 25.20% of samples (Table 4). The mean contamination levels of the AFB_1_ maize samples was 1.73 ppb (95% CI: 0.17–3.30) while the mean contamination level of the samples with total fumonisins was 0.66ppm (95% CI: 0.46–0.87). The mean moisture content of the maize samples was 9.70% (95% CI: 9.50%–13.40%). For eight tortilla makers, fumonisins were detected in the maize, tortilla, and urine samples.

**Table 4:** Summary table of the presence of mycotoxins in maize, tortilla, and urine samples.

Mycotoxin Presence	Maize Samples (n = 123)*	Tortilla Samples (n = 140)*	Urine Samples (n = 141)*
Fumonisin presence only (total fumonisins in maize and tortillas; uFB_1_ in urine)	64 (52.03%)	45 (32.14%)	17 (12.06%)
Aflatoxin presence only (AFB_1_)	3 (2.44%)	15 (10.71%)	-
Both total fumonisin and AfB_1_ presence	31 (25.20%)	2 (1.43%)	-
Fumonisin or aflatoxin presence not detected (total fumonisins in maize and tortillas; uFB_1_ in urine; AFB_1_ in maize and tortillas)	25 (20.33%)	78 (55.71%)	124 (87.94%)

*Of the 141 tortillerias, 18 did not have maize available to purchase from the tortilleria or from the source (123 maize samples collected) and one did not have tortillas to purchase (140 tortilla samples collected). The difference in the number of tortilla, maize, and urine samples is due to interviews taking place during a time of low activity of the tortilleria.

**Fig 2:**
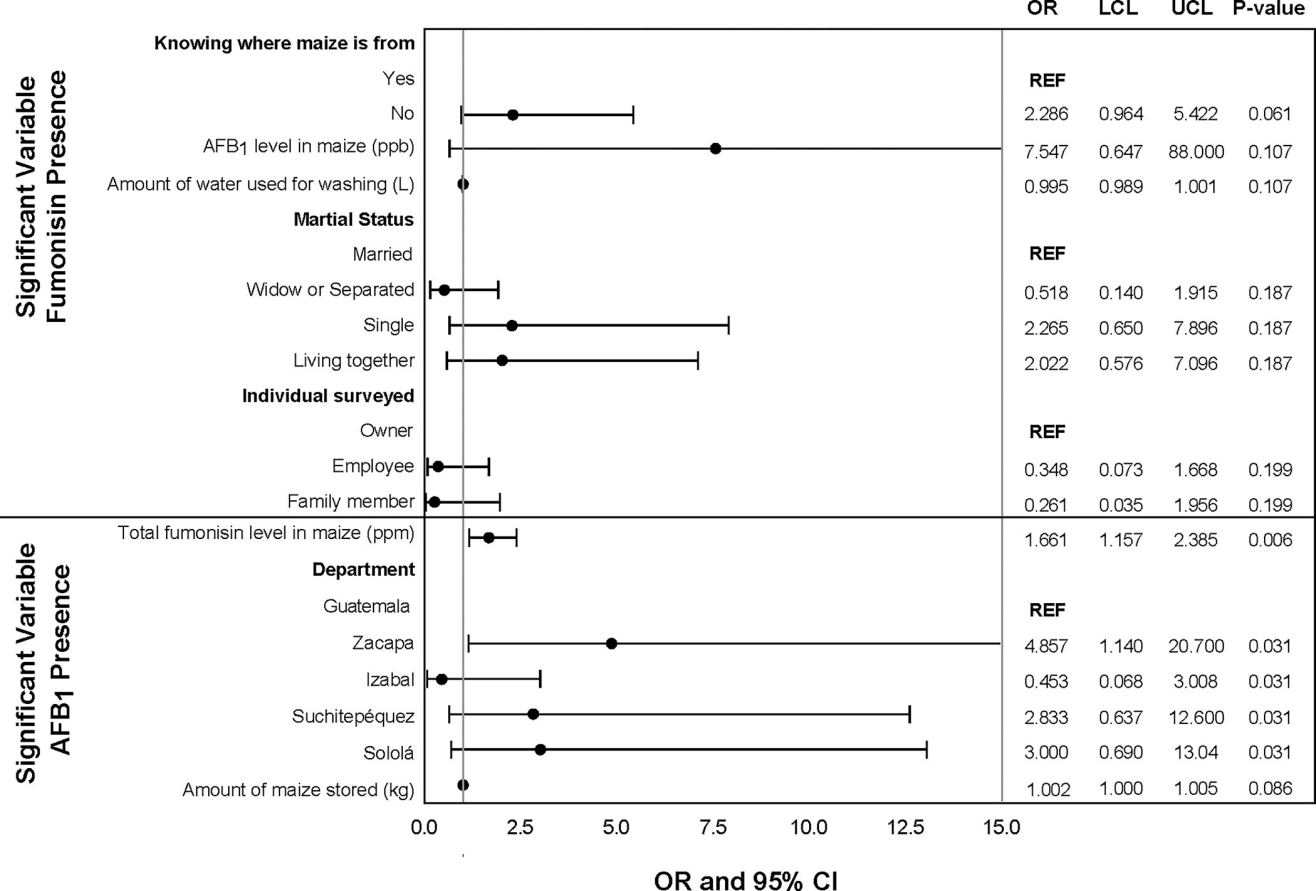
Univariate analysis of maize characteristics and handling practices associated with presence of AFB_1_ and total fumonisin in maize samples (p<0.2). Odds ratios (OR), upper (UCL) and lower (LCL) 95% confidence levels, and p-values from logistic regression.

**Fig 3:**
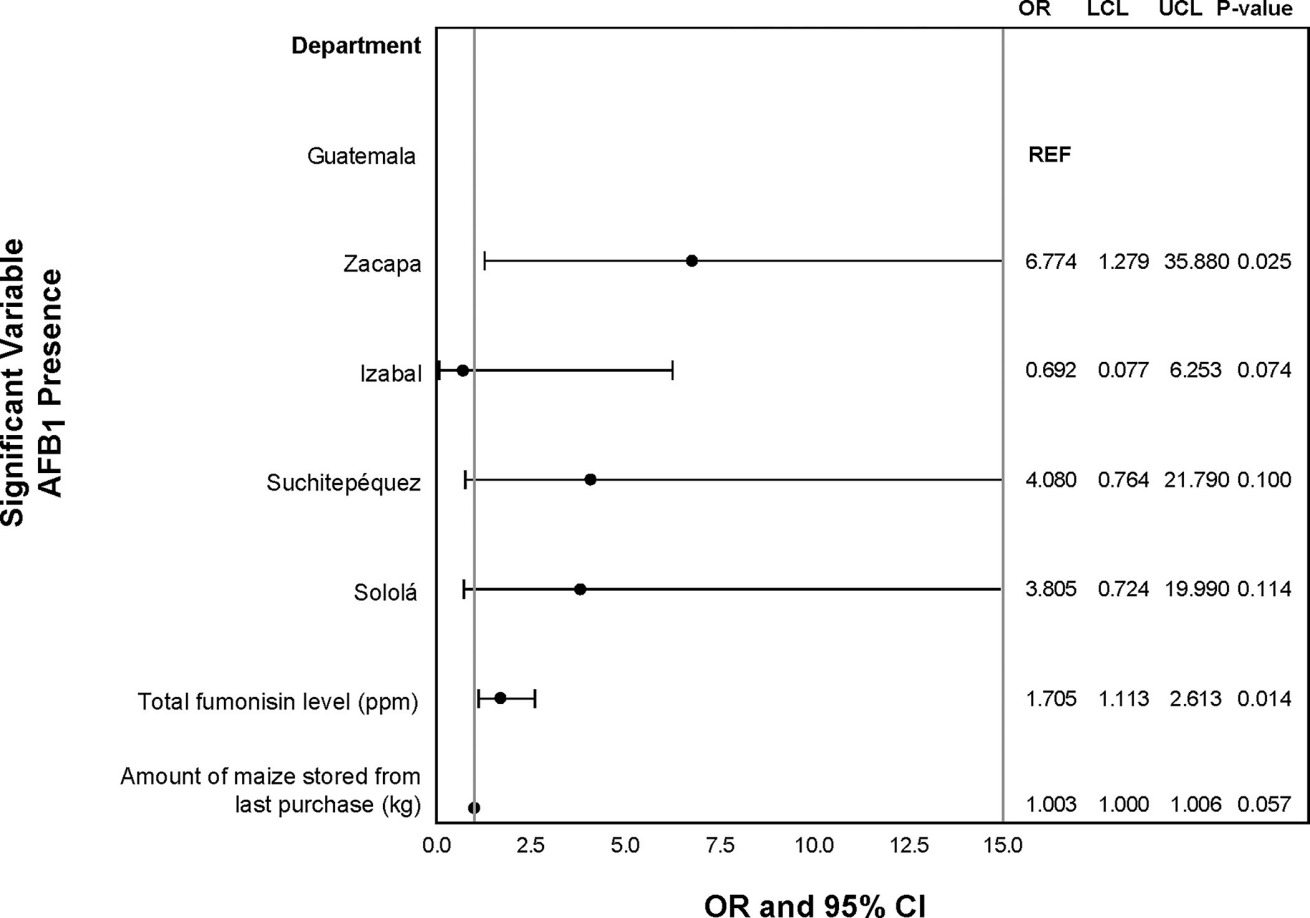
Multivariate analysis of sociodemographic characteristics, maize characteristics, and handling practices associated with presence of AFB_1_ in maize samples. Odds ratios (OR), upper (UCL) and lower (LCL) 95% confidence levels, and p-values from logistic regression.

Presence/absence of total fumonisins was marginally associated with AFB_1_ level, not knowing the source of the maize, and marital status in the univariable analysis. The level of AFB_1_ in maize was marginally significant in the univariate analysis for increasing risk of fumonisin presence in maize samples. Not being married was marginally associated with fumonisin presence in maize samples when compared with being married. Those who did not know were there maize was from were 2.3 times more likely to have fumonisins present in their maize samples compared to those who knew where their maize was from. This was a marginal association (Fig 2). The multivariate model for presence of total fumonisin level in maize samples did not converge. In both the univariate and multivariate analysis, the presence/absence of AFB_1_ in maize was associated with the total fumonisin level in the sample and the department the tortilleria was located in. The total fumonisin level in maize was associated in the univariate and multivariate analysis for increasing risk of AFB_1_ presence in maize samples (Fig 3). The amount of maize stored from the last purchase was marginally significant in the univariate and multivariate analysis for increasing risk of AFB_1_ presence in maize samples (Figs 2 and 3).

### Tortilla contamination and tortilla making practices

Of the 140 tortilla samples collected, 10.71% contained only AFB_1_, 32.14% contained only total fumonisins, and both aflatoxins and fumonisins were detected in 1.43% of samples (Table 4). The mean contamination level of the tortilla samples with AFB_1_ was 0.713 ppb (95% CI: 0.00–1.57), while the mean contamination level of the tortilla samples with total fumonisins was 0.040 ppm (95% CI: 0.021–0.058). These levels represent a 58.79% reduction in the mean AFB_1_ level and a 93.94% reduction in the total fumonisin levels from the maize samples.

**Fig 4:**
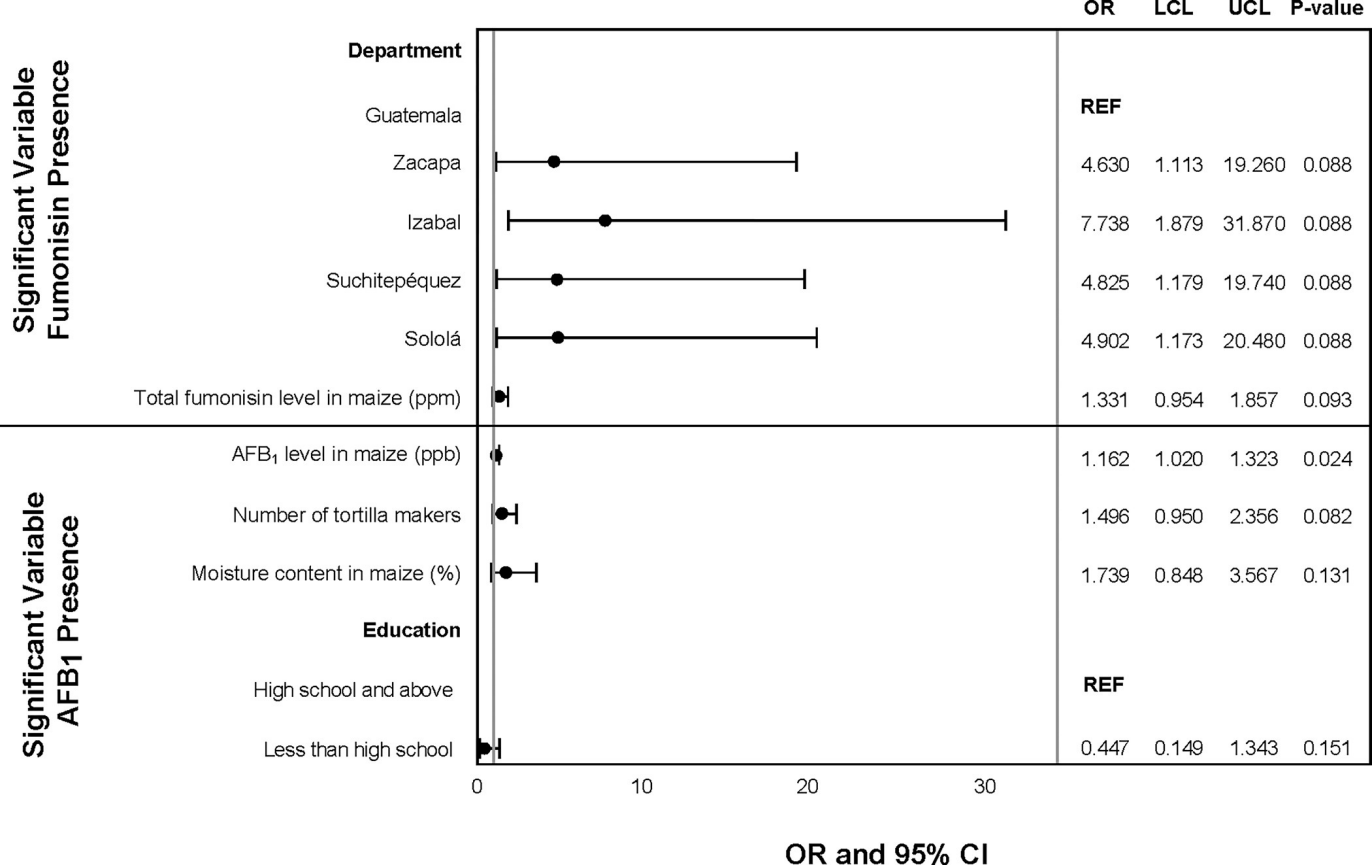
Univariate analysis of sociodemographic characteristics and tortilla preparation practices associated with presence of AFB_1_ and total fumonisin in tortilla samples (p<0.2). Odds ratios (OR), upper (UCL) and lower (LCL) 95% confidence levels, and p-values from logistic regression.

**Fig 5:**
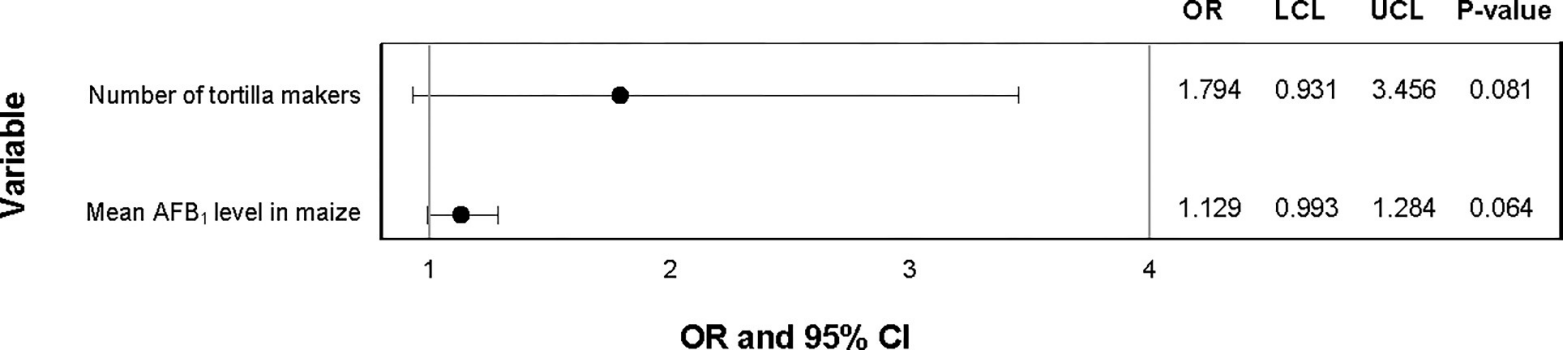
Multivariate analysis of sociodemographic characteristics and tortilla preparation practices associated with presence of AFB_1_ in tortilla samples. Odds ratios (OR), upper (UCL) and lower (LCL) 95% confidence levels, and p-values from logistic regression.

The association between presence/absence of total fumonisins in tortillas and total fumonisin level in maize was marginally significant (Fig 4). The department where the tortilleria was located was significantly associated with presence of total fumonisins in tortillas in both the univariate and multivariate models (Figs 4 and 5).

The association between presence/absence of AFB_1_ in tortillas and AFB_1_ level in maize samples was marginally significant (Fig 5). The number of tortilla makers in the tortilleria was significantly associated with presence of AFB_1_ (Fig 5). In the univariate analysis, the educational status of the individual and the moisture content of the maize was marginally associated with AFB_1_ presence in the tortillas (Fig 4).

Interaction effects between presence/absence of total fumonisins or AFB_1_ in maize and tortillas were evaluated, but not significant at an α = 0.20 level, so they were not included in the univariate model. The multivariate model for total fumonisin presence or absence in tortillas did not converge, thus only the univariable model was presented.

### Exposure

**Fig 6:**
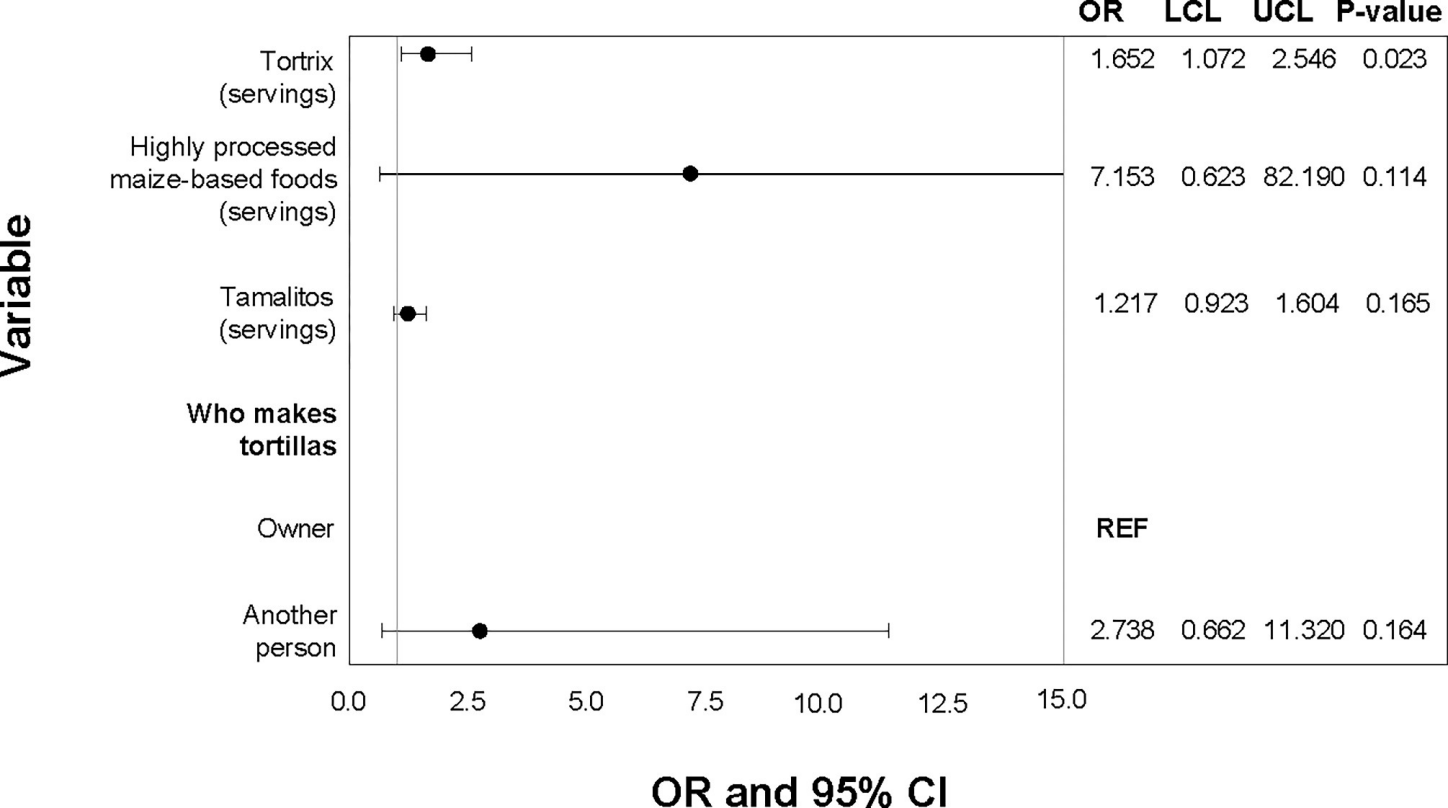
Univariate analysis of sociodemographic characteristics, maize handling practices, tortilla preparation practices, and dietary factors associated with presence of uFB_1_ in urine samples (p<0.2). Odds ratios (OR), upper (UCL) and lower (LCL) 95% confidence levels, and p-values from logistic regression.

Of the 141 urine samples, 12.06% tested positive for uFB_1_ and, thus, were considered exposed to FB_1_ (Table 3). Consuming an increased amount of Tortrix was significantly associated with FB_1_ exposure (Fig 6). Additionally, consumption of highly processed maize-based foods and tamalitos were marginally associated with FB_1_ exposure (Fig 6). AFB_1_ and total fumonisin levels in maize and tortillas were not significantly associated with exposure (AFB_1_ maize p = 0.308; AFB_1_ tortillas p = 0.562; total fumonisins maize p = 0.516, total fumonisins tortillas p = 0.261). The multivariate model for uFB_1_ presence or absence in urine only included Tortrix as a factor, thus the univariable model was presented.

## Discussion

The uFB_1_ levels found in this study exceeded 0.5 ng/mL in urine, which is the uFB_1_ level expected to approximate the provisional maximum tolerable daily intake set by the FAO/WHO Expert Committee on Food Additives of 2 μg/kg body weight (bw)/day (JECFA PMTDI) [18,22,42,47]. As the limit of detection in the present experiment was over 2 times this set level (at 1.15 ng/mL urine), the 17 women who were shown to test positive for Fumonisin B_1_ in their urine were considered highly exposed. There is a higher risk for adverse health outcomes if individuals consume amounts of fumonisins greater than the JECFA PMTDI [18].

Mycotoxin levels in tortillas were lower than in maize suggesting that nixtamalization reduced mycotoxin levels adding to a body of scientific evidence that builds upon cultural knowledge [48]. Nixtamalization processes have been shown to reduce aflatoxin and fumonisin levels 50–75% depending on the processing methods used [10,14,14,49]. In this study, a reduction in the AFB_1_ and total fumonisin levels was found during tortilla processing which is consistent with other research [10,14,49,50]. The alkaline soaking step in the nixtamalization process can reduce mycotoxin levels but reduction levels depend on the specific nixtamalization process being used, which is often related to different cultural practices [10,11,14,48,50–55]. For example, water used for nixtamalization can become contaminated with mycotoxins if the maize is contaminated with mycotoxins. If the water for nixtamalization is re-used, subsequent batches of masa can become contaminated with mycotoxins. Since maize handling practices varied significantly, more research on the range and frequency of maize handling and nixtamalization practices used in Guatemala is needed to develop interventions to reduce contamination levels.

As expected, department was significantly associated with mycotoxin contamination and exposure in this study. Mycotoxin levels in maize and exposure to mycotoxins has been found to vary throughout Guatemala [23,36,38,56]. In this study, maize from Zacapa, Suchitepéquez, and Sololá were more likely to be contaminated with aflatoxins than those from the Guatemala department. Additionally, tortillas from Zacapa, Izabal, and Sololá were more likely to be contaminated with fumonisins than those from the Guatemala department. This could be due to differences in the climates in these regions. For example, Zacapa and Suchitepéquez both have hot climates. Suchitepéquez also has a humid climate. A hot and humid climate is conducive to mycotoxin production if maize is not handled and stored properly [6,57,58]. Previous research has found similar trends for with high levels of fumonisin contamination in Zacapa (with 16/16 positive samples), Izabal (with 14/14 positive samples), as well as Sololá (with 4/6 positive samples) [56].

Staggered growing and harvesting seasons in Guatemala can result in exposing more individuals to mycotoxins if maize is contaminated. Due to its multiple different climate zones, Guatemala has multiple maize harvests per year [59]. Subsequently, the different regions in Guatemala have different growing seasons. As a result, maize purchasing practices change throughout the year to provide maize year-round to all of the departments in the country [60,61]. For example, maize from one department is often sold to fill demand for maize in another department. If the maize is contaminated before it is shipped, mycotoxin levels can increase during transportation and subsequent storage, resulting in individuals being exposed to higher mycotoxin levels.

Tortilleria characteristics were also associated with mycotoxin exposure. Increasing the number of individuals working in the tortilla shop increased the risk of tortilla samples being positive for aflatoxins. If someone other than the owner of the tortilla shop made tortillas, risk of an individual having urine sample positive for uFB_1_ increased. Potential reasons for this include that individual workers from a tortilleria may be from different socioeconomic statuses, cultures and/or have different tortilla making practices such as the amount of lime added during nixtamalization [51]. Additionally, martial status could be potentially related to differences in cultural practices that include differences in maize handling and storage or access to finances for purchasing higher quality maize.

The association between increased consumption of Tortrix and highly processed maize-based food consumption with exposure to uFB_1_ was surprising. Due to processing methods such as extrusion, these food items would not be expected to contain fumonisins [44]. However, consumption of these products may be confounded with socioeconomic status. As individuals increase their income and move out of poverty, an increase in consumption of salty snacks has been noticed. The ability to buy snacks such as Tortix could be seen as a status symbol as it is a more westernized food [62–64]. Therefore, there is also a potential for survey bias with the reporting of consumption of this food. There could have been a potential for overreporting of consumption of Tortrix due to this. The association between increasing socioeconomic status and exposure to mycotoxins needs to be further explored.

Controlling tortilla mycotoxin contamination starts in agricultural fields with good maize handling and continues on farms and during transportation with proper storage practices (e.g., promptly drying maize to a sufficient moisture content after harvest to prevent contamination, avoiding bird and other pest damage). Additionally, tortillerias can use practices to reduce tortilla contamination levels by purchasing good quality maize (e.g., not cracked or with visible signs of fungal contamination) from known suppliers that use good maize handling practices. During the tortilla making process, tortillerias can avoid cross-contamination of batches of masa by not re-using water used for nixtamalization. Further research is needed to determine the culturally appropriate tortilla making practices for each region that reduce mycotoxins to the furthest extent. Finally, currently policies win Guatemala only establish a limit for aflatoxins in maize, additional policies for the reduction of other mycotoxins such as fumonisins in maize could further reduce mycotoxin exposure.

There are multiple limitations of this study that should be acknowledged when results are interpreted. First, only one tortilla maker per tortilleria was asked how tortillas were prepared at that tortilleria, even though practices could vary within a tortilleria. Additionally, formal direct observation of the process of tortilla making was not conducted. Therefore, it is possible that the results are not representative of actual tortilleria practices. Future studies should examine variability of maize handling and tortilla making practices within tortillerias. Second, maize and tortilla samples were not collected from all tortillerias due to the timing of the surveys. Since missing data can impact results, sensitivity analyses were conducted; similar results were obtained with imputing these missing values as zero and the median value. Future studies should allocate more time in each study location so that all samples in a tortilleria could be collected. Third, due to limited resources, the dietary recall survey was limited to maize-based foods. As such, we were not able to assess diet diversity or consider other foods that may be contaminated with mycotoxins, such as cereal grains. Even so, maize-based foods have been shown to make up the majority of the Guatemalan diet [42,47,56]. Fourth, urine samples were taken on the day of the interview. The uFB_1_ biomarker demonstrates an individual’s exposure to FB_1_ over the past 24–48 hours [18,22] and, as such, may not be representative of their long-term exposure. This is important since diseases caused by mycotoxins are generally due to chronic exposure. The dietary patterns of Guatemalans have been shown to be consistent over time [42,47,56,62,64,65], so it is possible that the measured uFB_1_ levels are representative of long-term exposure. Future studies should evaluate an individual’s exposure over time. Fifth, due to limited resources, we were unable to quantify fumonisin levels in the urine samples. It would help to know an individual’s specific level of exposure. Future method development could increase the sensitivity for quantification of levels of uFB_1_ in urine samples. Sixth, urine samples were only evaluated for the presence or absence of uFB_1_ due to limited resources. Co-exposure to aflatoxins and fumonisins should be evaluated in future research.

Prompt adoption of these culturally appropriate mycotoxin mitigation strategies in areas of high mycotoxin contamination could lead to less mycotoxin production and decreased mycotoxin exposure. Results from this study can aid in the development of interventions and policies improve food safety and reduce exposure to mycotoxins.

## Supporting information

S1 Checklist Ethical, cultural, and scientific considerations specific to inclusivity in global research. (PDF)

S1 Material English version of survey used for this study. (PDF)

S2 Material Spanish version of survey used for this study. (PDF)

S1 Table Data dictionary for raw data for article including variable names, description, and units. (CSV)

S2 Table Raw data for article. (CSV)

## References

[1] Chen C, Riley RT, Wu F. Dietary Fumonisin and Growth Impairment in Children and Animals: A Review. Comprehensive Reviews in Food Science and Food Safety. 2018;17: 1448–1464. doi: 10.1111/1541-4337.12392 33350142

[2] Shirima CP, Kimanya ME, Routledge MN, Srey C, Kinabo JL, Humpf H-U, et al. A Prospective Study of Growth and Biomarkers of Exposure to Aflatoxin and Fumonisin during Early Childhood in Tanzania. Environmental Health Perspectives. 2015;123: 173–178. doi: 10.1289/ehp.1408097 25325363 PMC4314247

[3] Liu Y, Chang CCH, Marsh GM, Wu F. Population attributable risk of aflatoxin-related liver cancer: Systematic review and meta-analysis. European Journal of Cancer. 2012. doi: 10.1016/j.ejca.2012.02.009 22405700 PMC3374897

[4] Qian GS, Ross RK, Yu MC, Qian G, Ross RK, Yu MC, et al. A follow-up study of urinary markers of aflatoxin exposure and liver cancer risk in Shanghai, People ‘ s Republic of China. A Follow-Up Markers of China1. Cancer Research. 1994;3: 3–10. 8118382

[5] Guangxi S, Yeh F, Yu MC, Mo C, Luo S, Tong MJ, et al. Hepatitis B Virus, Aflatoxins, and Hepatocellular Carcinoma in. Cancer Research. 1989;49: 2506–2509. 2539905

[6] Wild CP, Miller JD, Groopman JD, editors. Mycotoxin control in low-and middle-income countries. Lyon, France: International Agency for Research on Cancer; 2015. Available from: https://publications.iarc.fr/Book-And-Report-Series/Iarc-Working-Group-Reports/Mycotoxin-Control-In-Low—And-Middle-income-Countries-2015. 27030861

[7] International Agency for Research on Cancer Working Group on the Evaluation of Carcinogenic Risks to Humans. Chemical Agents and Related Occupations. IARC Monographs on the Evaluation of Carcinogenic Risks to Humans, No. 100F. Lyon: International Agency for Research on Cancer; 2012. Available from: https://publications.iarc.fr/Book-And-Report-Series/Iarc-Monographs-On-The-Identification-Of-Carcinogenic-Hazards-To-Humans/Chemical-Agents-And-Related-Occupations-2012.

[8] Torres O, Matute J, Gelineau-van Waes J, Maddox JR, Gregory SG, Ashley-Koch AE, et al. Human health implications from co-exposure to aflatoxins and fumonisins in maize-based foods in Latin America: Guatemala as a case study. World Mycotoxin Journal. 2015;8: 143–159. doi: 10.3920/WMJ2014.1736

[9] Castillo-Urueta P, Carvajal M, Méndez I, Meza F, Gálvez A. Survey of aflatoxins in maize tortillas from Mexico City. Food Additives and Contaminants: Part B. 2011;4: 42–51. doi: 10.1080/19393210.2010.533390 24779661

[10] Elias-Orozco R, Castellanos-Nava A, Gaytán-Martínez M, Figueroa-Cárdenas JD, Loarca-Piña G. Comparison of nixtamalization and extrusion processes for a reduction in aflatoxin content. Food Additives and Contaminants. 2002;19: 878–885. doi: 10.1080/02652030210145054 12396399

[11] Méndez-Albores J, Villa G, Del Rio-García J, Martínez E. Aflatoxin-detoxification achieved with Mexican traditional nixtamalization process (MTNP) is reversible. J Sci Food Agric. 2004;84: 1611–1614. doi: 10.1002/jsfa.1853

[12] Price RL, Jorgensen KV. Effects of Processing on Aflatoxin Levels and on Mutagenic Potential of Tortillas made from Naturally Contaminated Corn. Journal of Food Science. 1985;50: 347–349. 10.1111/j.1365-2621.1985.tb13398.x.

[13] Torres P, Guzmán-Ortiz M, Ramírez-Wong B. Revising the Role of pH and Thermal Treatments in Aflatoxin Content Reduction During the Tortilla and Deep Frying Processes. J Agric Food Chem. 2001;49: 2825–2829. doi: 10.1021/jf0007030 11409972

[14] Palencia E, Torres O, Hagler W, Meredith FI, Williams LD, Riley RT. Total Fumonisins Are Reduced in Tortillas Using the Traditional Nixtamalization Method of Mayan Communities. The Journal of Nutrition. 2003;133: 3200–3203. doi: 10.1093/jn/133.10.3200 14519811

[15] Bowers J, Brown B, Springer J, Tollefson L, Lorentzen R, Henry S. Risk Assessment for Aflatoxin: An Evaluation Based on the Multistage Model. Risk Analysis. 1993;13: 637–642. doi: 10.1111/j.1539-6924.1993.tb01325.x 8310162

[16] Groopman JD, Kensler TW, Wild CP. Protective Interventions to Prevent Aflatoxin-Induced Carcinogenesis in Developing Countries. Annual Review of Public Health. 2008;29: 187–203. doi: 10.1146/annurev.publhealth.29.020907.090859 17914931

[17] Food and Agriculture Organization of the United Nations, World Health Organization. Codex Alimentarius International Food Standards General Standard for Contaminants and Toxins in Food and Feed. 2019. Report No.: CXS 193–1995. Available from: https://www.fao.org/fao-who-codexalimentarius/sh-proxy/en/?lnk=1&url=https%253A%252F%252Fworkspace.fao.org%252Fsites%252Fcodex%252FStandards%252FCXS%2B193-1995%252FCXS_193e.pdf

[18] World Health Organization & Joint FAO/WHO Expert Committee on Food Additives. Evaluation of certain contaminants in food: eighty-third report of the Joint FAO/WHO Expert Committee on Food Additives. Geneva, Switzerland: World Health Organization; 2017. Report No.: 1002. Available from: https://apps.who.int/iris/handle/10665/254893.

[19] Rheeder JP, Marasas WFO, Vismer HF. Production of Fumonisin Analogs by Fusarium Species. Appl Environ Microbiol. 2002;68: 2101–2105. doi: 10.1128/AEM.68.5.2101-2105.2002 11976077 PMC127586

[20] Bartók T, Szécsi Á, Szekeres A, Mesterházy Á, Bartók M. Detection of new fumonisin mycotoxins and fumonisin-like compounds by reversed-phase high-performance liquid chromatography/electrospray ionization ion trap mass spectrometry. Rapid Communications in Mass Spectrometry. 2006;20: 2447–2462. doi: 10.1002/rcm.2607 16871522

[21] Chen C, Mitchell NJ, Gratz J, Houpt ER, Gong Y, Egner PA, et al. Exposure to aflatoxin and fumonisin in children at risk for growth impairment in rural Tanzania. Environment International. 2018;115: 29–37. doi: 10.1016/j.envint.2018.03.001 29544138 PMC5989662

[22] Riley RT, Torres O, Matute J, Gregory SG, Ashley-Koch AE, Showker JL, et al. Evidence for fumonisin inhibition of ceramide synthase in humans consuming maize-based foods and living in high exposure communities in Guatemala. Mol Nutr Food Res. 2015;59: 2209–2224. doi: 10.1002/mnfr.201500499 26264677 PMC4956729

[23] Torres O, Matute J, Gelineau-van Waes J, Maddox JR, Gregory SG, Ashley-Koch AE, et al. Urinary fumonisin B _1_ and estimated fumonisin intake in women from high- and low-exposure communities in Guatemala. Mol Nutr Food Res. 2014;58: 973–983. doi: 10.1002/mnfr.201300481 24375966

[24] Rustom IYS. Aflatoxin in food and feed: occurrence, legislation and inactivation by physical methods. Food Chemistry. 1997;59: 57–67. doi: 10.1016/S0308-8146(96)00096-9

[25] Castelo MM, Sumner SS, Bullerman LB. Stability of Fumonisins in Thermally Processed Corn Products†. Journal of Food Protection. 1998;61: 1030–1033. doi: 10.4315/0362-028X-61.8.1030 9713766

[26] Schatzmayr G, Streit E. Global occurrence of mycotoxins in the food and feed chain: facts and figures. World Mycotoxin Journal. 2013;6: 213–222. doi: 10.3920/WMJ2013.1572

[27] Simões D, Carbas B, Soares A, Freitas A, Silva AS, Brites C, et al. Assessment of Agricultural Practices for Controlling Fusarium and Mycotoxins Contamination on Maize Grains: Exploratory Study in Maize Farms. Toxins. 2023;15: 136. doi: 10.3390/toxins15020136 36828450 PMC9964085

[28] Dombrink-Kurtzman MA, Dvorak TJ, Barron ME, Rooney LW. Effect of Nixtamalization (Alkaline Cooking) on Fumonisin-Contaminated Corn for Production of Masa and Tortillas. J Agric Food Chem. 2000;48: 5781–5786. doi: 10.1021/jf000529f 11087554

[29] Milani J, Maleki G. Effects of processing on mycotoxin stability in cereals: Mycotoxins in cereal processing. J Sci Food Agric. 2014;94: 2372–2375. doi: 10.1002/jsfa.6600 24497303

[30] Schaarschmidt S, Fauhl-Hassek C. Mycotoxins during the Processes of Nixtamalization and Tortilla Production. Toxins. 2019;11: 227. doi: 10.3390/toxins11040227 30995755 PMC6520960

[31] Ministerio de Salud Pública y Asistencia Social (MSPAS), Instituto Nacional de Estadística (INE), ICF International. Encuesta Nacional de Salud Materno Infantil 2014–2015. Guatemala; 2015. Report No.: Informe Final. Available from: https://www.dhsprogram.com/pubs/pdf/fr318/fr318.pdf.

[32] Castelino JM, Dominguez-Salas P, Routledge MN, Prentice AM, Moore SE, Hennig BJ, et al. Seasonal and gestation stage associated differences in aflatoxin exposure in pregnant Gambian women. Trop Med Int Health. 2014;19: 348–354. doi: 10.1111/tmi.12250 24372685 PMC4034353

[33] Mapesa JO, Maxwell AL, Ryan EP. An Exposome Perspective on Environmental Enteric Dysfunction. Environmental Health Perspectives. 2016;124: 1121–1126. doi: 10.1289/ehp.1510459 26713888 PMC4977058

[34] Turner PC, Collinson AC, Cheung YB, Gong Y, Hall AJ, Prentice AM, et al. Aflatoxin exposure in utero causes growth faltering in Gambian infants. International Journal of Epidemiology. 2007;36: 1119–1125. doi: 10.1093/ije/dym122 17576701

[35] Comisión Guatemalteca de Normas (COGUANOR). Maíz en grano. Maíz elaborado. NGO 34 047 Jul 5, 1982. Available from: https://cretec.org.gt/wp-content/uploads/2021/03/ngo34047maizelaborado.pdf

[36] Kroker-Lobos MF, Alvarez CS, Rivera-Andrade A, Smith JW, Egner P, Torres O, et al. Association between aflatoxin-albumin adduct levels and tortilla consumption in Guatemalan adults. Toxicology Reports. 2019;6: 465–471. doi: 10.1016/j.toxrep.2019.05.009 31193789 PMC6541741

[37] Mendoza JR, Rodas A, Oliva A, Sabillón L, Colmenares A, Clarke J, et al. Safety and Quality Assessment of Smallholder Farmers’ Maize in the Western Highlands of Guatemala. Journal of Food Protection. 2018;81: 776–784. doi: 10.4315/0362-028X.JFP-17-355 29624105

[38] Smith JW, Kroker-Lobos MF, Lazo M, Rivera-Andrade A, Egner PA, Wedemeyer H, et al. Aflatoxin and viral hepatitis exposures in Guatemala: Molecular biomarkers reveal a unique profile of risk factors in a region of high liver cancer incidence. Chemin I, editor. PLoS ONE. 2017;12: e0189255. doi: 10.1371/journal.pone.0189255 29236788 PMC5728519

[39] Jolly PE, Mazariegos M, Contreras H, Balas N, Junkins A, Aina IO, et al. Aflatoxin Exposure Among Mothers and Their Infants from the Western Highlands of Guatemala. Matern Child Health J. 2021;25: 1316–1325. doi: 10.1007/s10995-021-03151-1 33945085 PMC8404172

[40] Mendoza JR, Sabillón L, Martinez W, Campabadal C, Hallen-Adams HE, Bianchini A. Traditional maize post-harvest management practices amongst smallholder farmers in Guatemala. Journal of Stored Products Research. 2017;71: 14–21. doi: 10.1016/j.jspr.2016.12.007

[41] Voth-Gaeddert LE, Stoker M, Torres OR, Oerther DB. The influence of local market and household factors on aflatoxin presence in maize and symptoms of its exposure to children in Guatemala. International Journal of Environmental Health Research. 2020;30: 312–326. doi: 10.1080/09603123.2019.1594721 30897935

[42] Riley RT, Torres O, Showker JL, Zitomer NC, Matute J, Voss KA, et al. The kinetics of urinary fumonisin B1 excretion in humans consuming maize-based diets. Mol Nutr Food Res. 2012;56: 1445–1455. doi: 10.1002/mnfr.201200166 22815244 PMC3820424

[43] Garsow AV, Torres OR, Matute JA, Riley RT, Harris JR, Lamichhane AP, et al. Dietary and socioeconomic risk factors for fumonisin exposure among women of reproductive age in 18 municipalities in Guatemala from 2013 to 2014. PLOS Global Public Health. 2022;2. doi: 10.1371/journal.pgph.0000337 36962498 PMC10021672

[44] Instituto de Nutrición de Centro América y Panamá (INCAP). Tabla de Composición de Alimentos de Centroamérica. Guatemala: Instituto de Nutrición de Centro América y Panamá (INCAP)/Organización Panamericana de la Salud (OPS); 2012 Feb. Report No.: 2^a^. Edición. Available from: http://www.incap.int/mesocaribefoods/dmdocuments/TablaCAlimentos.pdf.

[45] Riley RT, Torres OA, Palencia E. International shipping of fumonisins from maize extracts on C18 sorbent. Food Addit Contam. 2006;23: 826–832. doi: 10.1080/02652030600699650 16807208

[46] Wall-Martínez HA, Ramírez-Martínez A, Wesolek N, Brabet C, Durand N, Rodríguez-Jimenes GC, et al. Risk assessment of exposure to mycotoxins (aflatoxins and fumonisins) through corn *tortilla* intake in Veracruz City (Mexico). Food Additives & Contaminants: Part A. 2019;36: 929–939. doi: 10.1080/19440049.2019.1588997 30977716

[47] Torres O, Matute J, Gelineau-van Waes J, Maddox JR, Gregory SG, Ashley-Koch AE, et al. Urinary fumonisin B _1_ and estimated fumonisin intake in women from high- and low-exposure communities in Guatemala. Mol Nutr Food Res. 2014;58: 973–983. doi: 10.1002/mnfr.201300481 24375966

[48] Cheetham D. Corn, Colanders, and Cooking: Early Maize Processing in the Maya Lowlands and Its Implications. In: Staller J, Carrasco M, editors. Pre-Columbian Foodways: Interdisciplinary Approaches to Food, Culture, and Markets in Ancient Mesoamerica. New York, NY: Springer; 2010. pp. 345–368. doi: 10.1007/978-1-4419-0471-3_14

[49] Voss K, Ryu D, Jackson L, Riley R, Gelineau-van Waes J. Reduction of Fumonisin Toxicity by Extrusion and Nixtamalization (Alkaline Cooking). J Agric Food Chem. 2017;65: 7088–7096. doi: 10.1021/acs.jafc.6b05761 28170235

[50] Méndez-Albores JA, Arámbula-Villa G, Loarca-Piña MG, González-Hernández J, Castaño-Tostado E, Moreno-Martínez E. Aflatoxins’ fate during the nixtamalization of contaminated maize by two tortilla-making processes. Journal of Stored Products Research. 2004;40: 87–94. doi: 10.1016/S0022-474X(02)00080-2

[51] Bressani. Chemistry, technology, and nutritive value of maize tortillas. Food Reviews International. 2009;6: 225–264. doi: 10.1080/87559129009540868

[52] Bressani R, Paz y Paz R, Scrimshaw NS. Corn Nutrient Losses, Chemical Changes in Corn during Preparation of Tortillas. J Agric Food Chem. 1958;6: 770–774. doi: 10.1021/jf60092a009

[53] Bressani R, Scrimshaw NS. Lime-Heat Effects on Corn Nutrients, Effect of Lime Treatment on in Vitro Availability of Essential Amino Acids and Solubility of Protein Fractions in Corn. J Agric Food Chem. 1958;6: 774–778. doi: 10.1021/jf60092a010

[54] Katz SH, Hediger ML, Valleroy LA. Traditional Maize Processing Techniques in the New World. Science. 1974;184: 765–773. doi: 10.1126/science.184.4138.765 17783464

[55] Pappa MR, de Palomo PP, Bressani R. Effect of lime and wood ash on the nixtamalization of maize and tortilla chemical and nutritional characteristics. Plant Foods Hum Nutr. 2010;65: 130–135. doi: 10.1007/s11130-010-0162-8 20369297

[56] Torres OA, Palencia E, de Pratdesaba LL, Grajeda R, Fuentes M, Speer MC, et al. Estimated Fumonisin Exposure in Guatemala Is Greatest in Consumers of Lowland Maize. The Journal of Nutrition. 2007;137: 2723–2729. doi: 10.1093/jn/137.12.2723 18029490

[57] Garsow AV, Mendez D, Kowalcyk BB, Torres OR. Evaluation of the impact of pre- and post-harvest maize handling practices on mycotoxin contamination on smallholder farms in Guatemala. World Mycotoxin Journal. 2021;15: 261–268. doi: 10.3920/WMJ2021.2701

[58] Meredith FI, Torres OR, de TEJADA SS, Riley RT, Merrill AH. Fumonisin B _1_ and Hydrolyzed Fumonisin B _1_ (AP _1_) in Tortillas and Nixtamalized Corn (Zea mays L.) from Two Different Geographic Locations in Guatemala. Journal of Food Protection. 1999;62: 1218–1222. doi: 10.4315/0362-028X-62.10.1218 10528731

[59] Tay K. Guatemala Grain and Feed Annual Corn. USDA Foreign Agricultural Service; 2018 Sep p. 17. Available from: https://apps.fas.usda.gov/newgainapi/api/report/downloadreportbyfilename?filename=Grain%20and%20Feed%20Annual_Guatemala%20City_Guatemala_9-14-2018.pdf.

[60] Famine Early Warning Systems Network, United States Agency for International Development. Guatemala—Production and Trade Flow Map, Season 2. In: FEWS NET [Internet]. Jan 2009 [cited 20 Jun 2022]. Available from: https://fews.net/central-america-and-caribbean/guatemala/production-and-trade-flow-map/january-2009-2.

[61] Famine Early Warning Systems Network, United States Agency for International Development. Production and Market Flow Map: Centeral America First Maize. In: FEWS NET Famine Early Warning Systems Network [Internet]. Available from: https://www.fews.net/sites/default/files/documents/reports/Central_America_maize_fullmap_primera_en.pdf.

[62] Bermudez OI, Tucker KL. Trends in dietary patterns of Latin American populations. Cad Saúde Pública. 2003;19: S87–S99. doi: 10.1590/s0102-311x2003000700010 12886439

[63] Ford ND, Jaacks LM, Martorell R, Mehta NK, Perrine CG, Ramirez-Zea M, et al. Dietary patterns and cardio-metabolic risk in a population of Guatemalan young adults. BMC Nutr. 2017;3: 68. doi: 10.1186/s40795-017-0188-5 29892467 PMC5993443

[64] Mayén A-L, Stringhini S, Ford ND, Martorell R, Stein AD, Paccaud F, et al. Socioeconomic predictors of dietary patterns among Guatemalan adults. Int J Public Health. 2016;61: 1069–1077. doi: 10.1007/s00038-016-0863-3 27421467 PMC5300047

[65] Bermudez OI, Hernandez L, Mazariegos M, Solomons NW. Secular Trends in Food Patterns of Guatemalan Consumers: New Foods for Old. Food Nutr Bull. 2008;29: 278–287. doi: 10.1177/156482650802900404 19227052

