# Supplementary material for: Dietary, socioeconomic, and maize handling practices associated with aflatoxin and fumonisin exposure among women tortilla makers in 5 departments in Guatemala
Source: PLOS Glob Public Health. 2024 Feb 7;4(2):e0001623. doi: 10.1371/journal.pgph.0001623 (PMC10849247; doi:10.1371/journal.pgph.0001623)
Supplement: S1 Material — (PDF) [file pgph.0001623.s002.pdf]

Supplemental Material

Dietary, socioeconomic, and maize handling practices associated with aflatoxin and fumonisin exposure among women tortilla makers in 5 departments in Guatemala

Ariel V. Garsow, Olga R. Torres, Jorge A. Matute, Danielle M. Voss, Gonzalo Miyagusuku-Cruzado, M. Monica Giusti, Barbara B. Kowalcyk

Cod Totilleria __ __ __

SURVEY TOOL -- Module 1: TORTILLERIA

"Estimating mycotoxin exposure and improving food safety in Guatemala"

(CIENSA, February 2022)

Study title: Estimation of mycotoxin exposure and increased food security in Guatemala
Researchers: Barbara Kowalcyk, Olga Torres, Ariel Garsow
Sponsors: Private Donor and The Ohio State University

Paste ID sticker HERE

I. General Information about the Tortilleria

**0.** Tortilleria ID ________________	
1. Department: ________________________ 2. Code _____	**Guatemala = 1, Sololá = 2, Suchitepéquez = 3, Izabal = 4, Zacapa = 5.**
3. Municipality __________________________ 4. Code ____	
5. Neighborhood/Colony__________________ 6. Code ____	
7. Longitude _______ 8. Latitude _______	9. Temperature ________ C 10. Humidity ___________ %
11. Time when the information for the previous variables ___ ___. ___ ___ **hours**	
12. Name of the tortilleria ___________________<br>14. Tortilleria owner name __________________	13. Address _____________________________<br>15. Cell phone number _____________________
16. Number of people working in tortilleria ___ ___ 1. Owner ___ 2. family ____ 3. employee ____	
17. Person who gives information about the tortilleria: 1. Owner:___ 2. family ____ 3. employee ____	
18. Name of the person interviewed ___________________________________ 19. Age ___ ___ **years**	
**20. Survey components:** 1. **Questionnaire ____ 2. corn sample ____**	
**3. Sample of tortillas ____ 4. Urine sample ____**	
**21.** Language interview was conducted: 1. **Spanish __ 2. Kaqchikel __ 3. Tz̈'utujil __ 4. K'ichë __ 5. Other __**<br>**22. Date ___ ___ /___ ___ / 2022 23. Interviewer name ______________________________ Cod ____**	

II. Conditions of the material of the tortilleria and storage of the corn

Site conditions or tortilleria (99 = Could not be observed)

Aspects to qualify	Tortilleria	Cod	Storage	Cod
**II. 1** Material that predominates on the floor?	________________	____	________________	____
**II. 2** Material that predominates on the walls?	________________	____	________________	____
**II. 3** Material that predominates in the ceiling?	________________	____	________________	____

III: Corn and tortilla making

Questions	Answer Options	Codes
**Origin, purchase of corn and storage**		
**III. 1** What do you/***do you all use*** to prepare the dough of the tortillas you make in this tortilla shop?	1. Corn only?<br>2. Only maseca?<br>3. Corn and maseca?<br>4. Corn and **1. Sorghum, 2. Rice, 3. barley 4. Other__________**	1 ____<br>2 ____<br>3 ____<br>4 ____
**III.2** What color is the corn you use/***Do you use*** to make tortillas?	1. White 2. Yellow 3: Black 4. Corinth<br>5. Other (E) ______________________________________	____

**III. 3** Almost always, what corn do you/***use*** to make tortillas is...	1. Harvested? , or ***-- (homegrown)---***<br>2. Purchased?<br>3. Other ***(specify)*** ____________________	____
**A. Harvested maize**		
**III. 4** Is corn harvested within the municipality?	1. Yes, ***Community ___________ Cod____***<br>0.No	____
	2. Don't know ➔Skip to question III.7	
**III. 5** Approximate distance from harvest community to tortilleria ___ ___ ***kilometers***		
**III.6** How do you transport harvested corn from the place where it is harvested to this place?	1. By land<br>2. By water (sea, lake, river)<br>3. Don't know/Don't respond	1 ____<br>2 ____<br>3 ____
**If the corn for tortillas is entirely homegrown, go to question III.12➔**		
**B. Maize purchased:**		
**III. 7** Here in _______________<br>Where do you buy/***buy*** mainly the corn you use/use to make the tortillas?	1. Producer's truck<br>2. Intermediary truck<br>3. Producer's boat<br>4. Intermediary boat<br>5. Warehouse/warehouse/market seller/terminal<br>6. Private shop/Deposit (outside of the market)<br>7. Shop/grocery store<br>8. Other ***(specify)*** __________________________	1 ____<br>2 ____<br>3 ____<br>4 ____<br>5 ____<br>6 ____<br>7 ____<br>8 ____
**III. 8** Do you know from which part of Guatemala comes the corn **that is bought** to make the masa with which tortillas are made?	1. Yes<br>0. Do not ➔ go to question III. 11<br>3. Don't Know/Don't Answer Move ➔ to Question Move to Question III.11	____
**III. 9** Where does the corn **that is bought** to make the tortillas come from?		

Answer Options	Codes	Answer Options	Codes
**1. South Coast**		**5. Peten**	
**2. North**		**6. Central Plateau**	
**3. East**		**7. Mexico**	
**4. The West**		**8. Other place *specify***	

**III. 10** How do you transport the corn that is bought to make the tortillas, from the place where it is harvested?	1. By land<br>2. By water (sea, lake, river)<br>3. Don't know/Don't respond	1 ____<br>2 ____<br>3 ____
**III. 11** Most of the time, the corn **that is bought** to make the tortillas...<br>***(Read answer options 1 and 2)***	1. Do you buy it daily?, ➔Skip to question III.20<br>2. Do you buy it for a longer time and keep it?<br>3. Use harvested corn	____

**III. 12** How long do you keep or store the corn before using it to make the tortillas?	1. Less than a week<br>2. One week<br>3. More than a week__ __***D / __ __ S / __ __ M***	____
**III.13** How do you store or store the corn you use to make these tortillas? ***(Read answer options 1 and 2)***	1. On the cob?, or<br>2. In grain?	____

III. 14 Where do you keep or store the corn you use to make tortillas?

Answer Options	Codes	Answer Options	Codes
**1. Troja**		**5. Silo**	
**2. Sack**		**6. Other**	
**3. Barrel / barrel**			

**III.15** Could I see where you keep the corn that you use/***use*** to make the tortillas?	1. Yes<br>2. The place is very secluded/locked<br>99. They declined observation ➔Move to question III.17	____

**Observation:** **III.16** What are the conditions of the place where this corn is stored?	**Aspects to observe:**	**YES**	**NO**
	1. Is it ventilated?	**1**	**0**
	2. It is illuminated: ***natural*** ___ ***artificial*** ____	**1**	**0**
	3. Is it wet?	**1**	**0**
	4. Is it clean?	**1**	**0**
	5. Is it used only to store corn?	**1**	**0**
	6. Completely closed / **Presence of holes? Yes __ No __**	**1**	**0**
	7. Are there pallets/ **If they are metal, are they rusty? Yes __ No __**	**1**	**0**

Quality of maize observed during the interview

**Observation:** **III.17** How is the presentation of the corn used to make the tortillas?	1. Whole<br>2. Broken<br>3. Chopped<br>4. With deteriorated natural color<br>5. Rotten<br>6. Other ***(specify)*** ______________________________	1 ____<br>2 ____<br>3 ____<br>4 ____<br>5 ____<br>6 ____

**Observation:** **III.18** Does the corn used to make tortillas have contaminants or foreign materials?	**Aspects to observe:**	**Quantity**	
	1. It doesn't have, it's clean		
	2. Roots, leaves, garbage	____	
	3. Stones, sticks	____	
	4. Weevils	____	
	5. Earth, dust, garbage	***Present*** ____ ***absent*** ____	
	6. Other (E) _____________	____	

**Observation:** **III.19** Does the corn used to make tortillas have any strong or noticeable odors?	1. It does not have<br>2. A fertilizer/herbicide/fungicide<br>3. A stale?<br>4. A rotten?<br>5. Other ***(specify)*** ________________________	1 ____<br>2 ____<br>3 ____<br>4 ____<br>5 ____

III. 20 How long ago was the **last time** you **bought** /***bought*** corn to make the tortillas you sell in this place***? 1)*** ___ ___ ***days 2)*** ___ ___ ***weeks 3)*** ____ ***NS/NR ➔Skip to question 6) of this III.20***

4) Do you know where the corn you bought last time came from***?***	***1***	***2***	***3***	***4***	***5***	***6***	***7***	***8***	***NS***

5) Did you keep corn from what you bought last time? 1. Yes __ 2. No __ ➔ P III. 21 | ***Quantity / Unit:*** ___ ___ / ____

6) How much corn is stored from the one you bought last time?	***Quantity / Unit: ___ ___ / ____***
**III.21 How** was the corn that you bought this last time to make the tortillas? ***(Read answer options, on each item)***	
a. 1. Whole?, 2 broken?, 3. picado?, or 4. rotten?	___ / ___ / ___ / ___
b. 1. Clean?, 2. con roots, 3. con stones, or earth, or with 4. weevils?	___ / ___ / ___ / ___
c. 1. With the smell of fertilizers/herbicides?, 2. Rancid, or smelling 3. rotten?	___ / ___ / ___
d. 99. Didn't review it / Doesn't remember	____

**Preparation of tortillas**		
**III. 22** What do you do when ***you*** realize that the corn to prepare the tortillas has: fungal weevils, or is eaten by birds or mice?	1. Discarded/Thrown Away<br>2. It is given to animals ***(pigs, poultry)***<br>3. Nothing, always used<br>4. Mix it with corn that has no harm<br>5. She /***they*** always buy good quality corn<br>6. Other ***(specify)*** ______________________	____

**III.23** Who is in charge of making **the nixtamal para** the dough with which they make the tortillas? ***(Ask the following question to the person in charge of preparing the nixtamal)***	1. The owner of the tortilleria<br>2. Another person<br>3. **They always buy the ready-made dough** ➔ ***Ask for THE KNOWLEDGE of the nixtamalization process)***	____
**III. 24** What are **the steps** you do/***what do you*** do to make the dough with which the tortillas are prepared, since the corn is obtained? ***(Read answer*** options ***1 to 6)*** ***Do you know what the steps are... ?***	____ Do you soak in lime water, overnight?<br>____ Is it boiled with lime (Nixtamalized)?<br>____ Boils without lime?<br>____Drains?<br>____ Is it washed several times with different water? __ ___ ***times***<br>____Is ground? (at home or in the motor mill)	1 ____<br>2 ____<br>3 ____<br>4 ____<br>5 ____<br>6 ____
	7. Doesn't know	7 ____

**III. 25** How much corn, water and lime do ***you use*** to make tortilla dough? ***Do you know how much...?***	**Ingredients (per grind)**	**Quantity**	
	1. Corn	**___ ___ lb/a**	
	2. Water for nixtamal	**___ ___ lt / bot / gal**	
	3. Lime	**___ ___ oz / lb**	
-------------------------------------- **III. 25a** How long is corn boiled?	4. Water for washing	**___ ___ lt / bot / gal**	
	5. Boiling time	**___ ___ h/m**	
--- ***Do you know how long...?***	***6. Don't know***		
	**How many torteadas do you do a day? ___ ___ torteadas**<br>**How many tortillas are made in C/torteada? ___ ___ ___ ___ tortillas**<br>**How many tortillas are made per day? ___ ___ ___ ___ tortillas**		

III.26 What kind of fuel do you/***Do you use*** to prepare the mase and make the tortillas?

**Answer Options**	**Codes**	**Answer Options**	**Codes**
**1. Firewood**		**4. Electricity**	
**2. Coal**		**5. Other (E)**	
**3. Propane gas**			

III. 27 Where do they get the water they use to make tortilla dough?

**Answer Options**	**Codes**	**Answer Options**
**1. Own jet**		**4. Public/relative or neighbor well**
**2. Public jet / relative or neighbor**		**5. River, lake, spring**
**3. Own well**		**Other (E)**

**III. 28** What do you/***do*** with the water you use for nixtamal and washing?<br>--<br>***What would you do with....?***	1. Use it again to soak, nixtamalize or drain<br>2. They use it to prepare other foods<br>3. They give it to animals ***(pigs, poultry)***<br>4. They throw it away or discard it<br>5. Other ***(specify)*** ______________________________	1 ____<br>2 ____<br>3 ____<br>4 ____<br>5 ____
	6. Don't know	
**III.29** After preparing the tortillas, what do you ***do*** with the dough that is left over?<br>--<br>***What would usted/ do you do...?***	1 They keep it to make tortillas of the next time/day<br>2. They use it for other preparations<br>3. Discard it thrown away or give to animals<br>4. Makes dough atol / fresh dough<br>5. Other ***(specify)*** ______________________________	1 ____<br>2 ____<br>3 ____<br>4 ____<br>5 ____
	6. Doesn't know	6 ____

Cod Totilleria __ __ __

SURVEY TOOL -- Module 2: WOMEN
"Estimating mycotoxin exposure and improving food safety in Guatemala"
(CIENSA, February 2022)

Study title: Estimation of mycotoxin exposure and increased food security in Guatemala

Researchers: Barbara Kowalcyk, Olga Torres, Ariel Garsow

Sponsors: Private Donor and The Ohio State University

Paste ID sticker HERE

I. General information of the woman interviewed

**0.** Tortilleria ID ____	
1. Department: ____________________ Code _____	**Guatemala = 1, Sololá = 2, Suchitepéquez = 3, Izabal = 4, Zacapa = 5.**
3. Municipality: ________________ Code ___ ___	
5. Neighborhood/Colony ________________________________ Code ___ ___	
7. Name of the tortilleria ________ 8. Code______	9. Cell phone number _______________________
10. Roles of the people who work in the tortilleria : 1. Owner ___ 2. family ____ 3. employee ____	
11. Selected person who gives information of **this survey**: 1. Owner:___ 2. family member ___ employee ____	
12. Name of interviewee ______________________________ Age ___ ___ years<br>***[Select this woman from those who want to participate in this study and give a urine sample]***	
14. Language in which the interview was conducted: **1. Español___ 2. Kaqchikel___ 3. Tź utujil___ 4. K̇ichë___ 5. Other ___**<br>**15. Date ___ ___ /___ ___ / 2022 16. Interviewer ______________________ Cod ____**	

II. Characteristics of the woman interviewed and her home

**Questions**		**Answer Options**				**Codes**
**II.1 Marital status of the interviewee**		***1. Married 2. United 3. Single 4. Widow 5. Divorced 6. Separate***				
**II. 2 Formal education of the woman interviewed**	***Can you read? ___<br>Do you know how to write? ___***	___________<br>***Degree***	____<br>***Code***	______________<br>***Level***		____<br>***Code***
**II. 3** Where are you?, Where were you born?	Community ______________________<br>Municipality ______________________<br>Department______________________					Code ___ ___ ___<br>Code ___ ___ ___<br>Code ___ ___
**II.4** How long ago did you leave your community?		**___ ___ months ___ ___ years**				

Part III. Consumption of prepared corn-based foods, during the last week

	**Did you eat ____ / Did you drink ____ last week?**				*Note: Consider all foods consumed by the target person, both at home and outside of the home, regardless of where they were prepared.*		
		**If you ate it or drank it...**					
**No**	**Food:**	**1 = Yes 0 = No**	**How many days a week did you eat/drink?**	**How much ____ Did you eat/drink in these ____days?**	**Units**		**Grams**
1	Tortillas	1 0	____	___ ___ ___. ___	or		40
2	Little Tamales	1 0	____	___ ___ ___. ___	or		200
3	Tamales	1 0	____	___ ___ ___ . ___	or		55
4	Chuchitos	1 0	____	___ ___ ___. ___	or		75
5	Sleeps	1 0	____	___ ___ ___. ___	or		65
6	Toast	1 0	____	___ ___ ___. ___	or		25
7	Nachos	1 0	____	___ ___ ___. ___	u/b		5 / 60
8	Tortrix	1 0	____	___ ___ ___. ___	b		40
9	Pinol	1 0	____	___ ___ ___. ___	cd/oz/lb /tz		8 /28. 7 / 454 / 95
10	Tayuyos / Tones	1 0	____	___ ___ ___. ___	or		55
11	Mass water	1 0	____	___ ___ ___. ___	v / tz / C		20 / 20/ 20
12	Mass atol	1 0	____	___ ___ ___. ___	v / tz / C		40 / 40 / 40
13	Corn coffee	1 0	____	___ ___ ___. ___	cd/ v /tz		12 /12 /12
14	Corn atol	1 0	____	___ ___ ___. ___	v / tz / C		40 / 40 / 40
15	Corn *(roasted/cooked)*	1 0	____	___ ___ ___. ___	or		115
16	Cornstarch	1 0	____	___ ___ ___. ___	cd /oz/lb/	tz	12 /28.7 /454 / 12
17	Polenta (CSB)	1 0	____	___ ___ ___. ___	cd /oz/lb/	tz	14 /28.7 /454 / 14
18	Corn flakes	1 0	____	___ ___ ___. ___	cd / tz		7 / 45
19	Incaparina	1 0	____	___ ___ ___. ___	cd / oz / lb-b / bp	tz	14 /28.7 /454 / 75 14
20	Tacos	1 0	____	___ ___ ___. ___	or		35
21	Other food	1 0	____	___ ___ ___. ___	u/cd /oz /lb/____		______

Unit abbreviations			
CD	= tablespoon	b	= bag
C	= ladle	BP	= sachet *(small bag)*
or	= unit	tz	= cup
Oz	= ounce	v	= glass
Lb	= pound		
