# Supplementary material for: Dietary, socioeconomic, and maize handling practices associated with aflatoxin and fumonisin exposure among women tortilla makers in 5 departments in Guatemala
Source: PLOS Glob Public Health. 2024 Feb 7;4(2):e0001623. doi: 10.1371/journal.pgph.0001623 (PMC10849247; doi:10.1371/journal.pgph.0001623)
Supplement: S2 Material — (PDF) [file pgph.0001623.s003.pdf]

Supplemental Material

Dietary, socioeconomic, and maize handling practices associated with aflatoxin and fumonisin exposure among women tortilla makers in 5 departments in Guatemala

Ariel V. Garsow, Olga R. Torres, Jorge A. Matute, Danielle M. Voss, Gonzalo Miyagusuku-Cruzado, M. Monica Giusti, Barbara B. Kowalcyk

Cod Tortillería __ __ __

INSTRUMENTO DE RECOLECCIÓN DE DATOS -- Módulo 1: TORTILLERÍA

"Estimando la exposición a micotoxinas y mejorando la inocuidad alimentaria en Guatemala"

(CIENSA, febrero de 2022)

Título del estudio: Estimación de exposición a micotoxinas e incremento de la seguridad alimentaria en Guatemala
Investigadores: Barbara Kowalcyk, Olga Torres, Ariel Garsow
Patrocinadores: Donante privado y The Ohio State University

Pegar calcomanía de ID AQUÍ

I. Información general de la tortillería

**0.** ID de la tortillería ____________________	
1. Departamento: ________________________________ 2. Código _____	**Guatemala = 1, Sololá = 2, Suchitepéquez = 3, Izabal = 4, Zacapa = 5.**
3. Municipio _____________________________________ 4. Código ____	
5. Barrio/Colonia__________________________________ 6. Código ____	
7. Longitud ____________ 8. Latitud ____________	9. Temperatura ________ C 10. Humedad __________ %
11. hora en que se tomó la información de las variables anteriores ___ ___ . ___ ___ **horas**	
12. Nombre de la tortillería ________________________________	13. Dirección ________________________
14. Nombre de la dueña de la tortillería _______________________	15. Celular _______________________
16. Número de personas que trabajan en la tortillería ___ ___ 1. Dueña ___ 2. familiar ____ 3. empleada ____	
17. Persona que da información de la tortillería: 1. Dueña:___ 2. familiar ____ 3. empleada ____	
18. Nombre de la persona entrevistada ____ **19.** Edad ___ ___ **años**	
**20. Componentes de la encuesta:** 1. **Cuestionario** ____ **2. muestra de maíz** ____	
**3. Muestra de tortillas** ____ **4. Muestra de orina** ____	
**21.** Idioma en la que se realizó la entrevista: 1. **Español** ___ **2. Kaqchikel** ___ **3. Tz¨utujil** ___ **4. K¨iche¨**___ **5. Otro** _____	
**22. Fecha** ___ ___ **/**___ ___ **/ 2022** **23. Encuestador/a** ______________________________ **24. Cod** ____	

II. Condiciones de la vivienda de la tortillería y almacenamiento del maíz

Condiciones del lugar o tortillería (99 = No se pudo observar)

Aspectos a calificar	Tortillería	Cod	Almacenamiento	Cod
**II.1** ¿Material que predomina en el piso?	________________	____	________________	____
**II.2** ¿Material que predomina en las paredes?	________________	____	________________	____
**II.3** ¿Material que predomina en el techo?	________________	____	________________	____

III: Maíz y elaboración de tortillas

Preguntas	Opciones de respuesta	Códigos
**Procedencia, compra de maíz y almacenamiento**		
**III.1** ¿Qué usa usted/***usan ustedes*** para preparar la masa de las tortillas que hacen en esta tortillería?	1. Solo maíz?<br>2. Solo maseca?<br>3. Maíz y maseca?<br>4. Maíz y **1. Maicillo, 2. Arroz, 3. cebada 4. Otro____________**	1 ____<br>2 ____<br>3 ____<br>4 ____
**III.2** ¿De qué color es el maíz que usa usted/***Usan ustedes*** para hacer las tortillas?	1. Blanco 2. Amarillo 3: Negro 4. Corinto<br>5. Otro (E) ___________________________________	____

Preguntas	Opciones de respuesta	Códigos
**III.3** Casi siempre, ¿El maíz qué usa usted/***usan ustedes*** para hacer las tortillas es …	1. Cosechado? , o ***-- (cosecha propia)---***<br>2. Comprado?<br>3. Otro ***(especifique)*** __________________________________	____
**A. Maíz cosechado**		
**III.4** ¿El maíz es cosechado dentro del municipio?	1. Si, ***Comunidad*** _______________________________ ***Cod*** ____<br>0. No<br>2. No sabe ➔Pase a pregunta III.7	____
**III.5** Distancia aproximada desde comunidad de cosecha a la tortillería ___ ___ ***kilómetros***		
**III.6** ¿Cómo transportan el maíz cosechado, desde el lugar donde se cosecha hasta este lugar?	1. Por tierra<br>2. Por agua (mar, lago, río)<br>3. No sabe/No responde	1 ____<br>2 ____<br>3 ____
**Si el maíz para las tortillas es totalmente de cosecha propia, ➔pase a pregunta III.12**		
**B. Maíz comprado:**		
**III.7** Aquí en ________________________ ¿Dónde compra usted/***compran ustedes*** principalmente el maíz que usa/usan para hacer las tortillas?	1. Camión del productor<br>2. Camión de intermediario<br>3. Lancha del productor<br>4. Lancha de intermediario<br>5. Bodega/depósito/vendedor de mercado/terminal<br>6. Bodega particular/Depósito (fuera de mercado)<br>7. Tienda/abarrotería<br>8. Otro ***(especifique)*** _________________________________	1 ____<br>2 ____<br>3 ____<br>4 ____<br>5 ____<br>6 ____<br>7 ____<br>8 ____
**III.8** ¿Sabe usted de que parte de Guatemala viene el maíz **que se compra** para hacer la masa con que se hacen las tortillas?	1. Si<br>0. No ➔ Pase a pregunta III. 11<br>3. No sabe/No responde Pase a pregunta ➔ Pase a pregunta III.11	____

III.9 ¿De dónde viene el maíz **que se compra** para hacer las tortillas?

Opciones de respuesta	Códigos	Opciones de respuesta	Códigos
**1. Costa sur**		**5. Peten**	
**2. Norte**		**6. Meseta Central**	
**3. Oriente**		**7. México**	
**4. Occidente**		**8. Otro lugar *especifique***	

**III.10** ¿Cómo transportan el maíz que se compra para hacer las tortillas, desde el lugar donde se cosecha?	1. Por tierra<br>2. Por agua (mar, lago, río)<br>3. No sabe/No responde	1 ____<br>2 ____<br>3 ____

**III.11** La mayoría de las veces, el maíz **que se compra** para hacer las tortillas… ***(Lea opciones de respuesta 1 y 2)***	1. Lo compran a diario?, ➔Pase a pregunta III.20<br>2. Lo compran para un mayor tiempo y lo guardan?<br>3. Usa maíz cosechado	____
**III.12** Por cuanto tiempo guarda o almacena usted/***usan ustedes*** el maíz antes de usarlos para hacer las tortillas?	1. Menos de una semana<br>2. Una semana<br>3. Más de una semana___ ___***D / ___ ___ S / ___ ___ M***	____
**III.13** ¿Cómo guarda o almacena usted/***almacenan ustedes*** el maíz que usan para hacer estas tortillas? ***(Lea opciones de respuesta 1 y 2)***	1. En mazorca?, o<br>2. En grano?	____

III.14 ¿Dónde guarda o almacena usted/Ustedes el maíz que usan para hacer las tortilla?

Opciones de respuesta	Códigos	Opciones de respuesta	Códigos
**1. Troja**		**5. Silo**	
**2. Costal**		**6. Otro**	
**3. Tonel / barril**			

**III.15** ¿Podría ver donde guardan el maíz que usa usted/***usan ustedes*** para hacer las tortillas?	1. Si<br>2. El lugar está muy retirado/cerrado con llave<br>**99.** No accedieron a la observación ➔Pase a pregunta III.17	____

	Aspectos a observar:	SI	NO
**Observación:** **III.16** ¿Cuáles son las condiciones que presenta el lugar donde se almacena este maíz?	1. Esta ventilado?	**1**	**0**
	2. Está iluminado: ***natural*** ___ ***artificial*** ____	**1**	**0**
	3. Está húmedo?	**1**	**0**
	4. Se encuentra limpio?	**1**	**0**
	5. Se usa solamente para guardar el maíz?	**1**	**0**
	6. Completamente cerrado / **Presencia de hoyos? Si __ No __**	**1**	**0**
	7. Hay tarimas / **Si son de metal, están oxidadas? Si __ No __**	**1**	**0**

Calidad del maíz observado durante la entrevista

**Observación:** **III.17** ¿Cómo es la presentación del maíz que se usa para hacer las tortillas?	1. Entero<br>2. Quebrado<br>3. Picado<br>4. Con color natural deteriorado<br>5. Podrido<br>6. Otro ***(especifique)*** ______________________________	1 ____<br>2 ____<br>3 ____<br>4 ____<br>5 ____<br>6 ____

	Aspectos a observar:	Cantidad	
**Observación:** **III.18** ¿Tiene contaminantes o materiales extraños, el maíz que se usa para hacer las tortillas?	1. No tiene, está limpio		
	2. Raíces, hojas, basura	____	
	3. Piedras, palos	____	
	4. Gorgojos	____	
	5. Tierra, polvo, basura	***Presente*** ____ ***ausente*** ____	
	6. Otro (E) _______________	____	

**Observación:** **III.19** ¿Tiene algún olor fuerte o perceptible, el maíz que se usa para hacer las tortillas?	1. No tiene<br>2. A fertilizante/herbicida/fungicida<br>3. A rancio?<br>4. A podrido?<br>5. Otro ***(especifique)*** ______________________________	1 ____<br>2 ____<br>3 ____<br>4 ____<br>5 ____

III.20 ¿Hace cuánto fue la **última vez** que usted **compró** /***compraron*** maíz para hacer las tortillas que vende en este lugar***? 1) ___ ___ días 2) ___ ___ semanas 3) ____ NS/NR ➔Pase a pregunta 6) de esta III.20***

4) ¿Sabe de dónde viene el maíz que compró está última vez?	***1***	***2***	***3***	***4***	***5***	***6***	***7***	***8***	***NS***
5) ¿Guardó maíz del que compró esta última vez? 1. Si ___ 2. No ___ ➔ P III. 21	***Cantidad / Unidad: ___ ___ / ____***								
6) ¿Qué cantidad de maíz se encuentra guardado del que compró la última vez?	***Cantidad / Unidad: ___ ___ / ____***								

III.21 ¿Cómo estaba el maíz que compró usted esta última vez para hacer las tortillas?
(Lea opciones de respuesta, en cada ítem)

a. 1. Entero?, 2 quebrado?, 3. picado?, ó 4. podrido?	___ / ___ / ___ / ___
b. 1. Limpio?, 2. con raíces, 3. con piedras, o tierra, o con 4. gorgojos?	___ / ___ / ___ / ___
c. 1. Con olor a fertilizantes/herbicidas?, 2. Rancio, o con olor a 3. podrido?	___ / ___ / ___
d. 99. No lo revisó / No se acuerda	____

**Preparación de tortillas (torteado)**		
**III.22** ¿Qué hace usted/***ustedes*** cuando se da cuenta que el maíz para preparar las tortillas tiene: gorgojos hongos, o está comido por pájaros o ratones?	1. Se descarta/Se tira<br>2. Se le da a los animales ***(cerdos, aves de corral)***<br>3. Nada, siempre se usa<br>4. Lo mezcla con maíz que no tiene daño<br>5. Ella /***ellas*** siempre compra maíz de buena calidad<br>6. Otro ***(especifique)*** ________________________________	____

**III.23** ¿Quién se encarga de hacer **el nixtamal p**ara la masa con que hacen las tortillas?<br>***(Haga la siguiente pregunta a la persona que se encarga de preparar el nixtamal)***	1. La dueña de la tortillería<br>2. Otra persona<br>3. **Siempre compran la masa ya preparada ➔ *Pregunte por EL CONOCIMIENTO del proceso de nixtamalización)***	____
**III.24** ¿Cuáles son **los pasos** que hace usted/***que hacen ustedes*** para hacer la masa con que se preparan las tortillas, desde que se consigue el maíz?<br>***(Lea opciones de respuesta 1 a 6)***	____ Se remoja en agua cal, durante la noche?<br>____ Se hierve con cal (Nixtamalizado)?<br>____ Se hierve sin cal?<br>____Se desagua?<br>____ Se lava varias veces con diferente agua? ___ ___ ***veces***<br>____Se muele? (en casa o en el molino de motor)	1 ____<br>2 ____<br>3 ____<br>4 ____<br>5 ____<br>6 ____
¿Sabe usted cuales son los pasos…?	***7. No sabe***	7 ____

	**Ingredientes** **(por molida)**	**Cantidad**	
**III.25** ¿Qué cantidad de maíz, agua y cal usa usted /***usan ustedes*** para hacer las masa de las tortillas?<br>***¿Sabe usted que cantidad…?***	1. Maíz	___ ___ **lb / a**	
	2. Agua para el nixtamal	___ ___ **lt / bot / gal**	
	3. Cal	___ ___ **oz / lb**	
---<br>**III.25a** ¿Por cuánto tiempo se hierve el maíz?	4. Agua para el lavado	___ ___ **lt / bot/ gal**	
	5. Tiempo de hervido	___ ___ **h / m**	
--<br>***¿Sabe usted por cuanto tiempo…?***	***6. No sabe***		
	**¿Cuántas torteadas hacen al día? ___ ___ torteadas**<br>**¿Cuántas tortillas se hacen en C/torteada? ___ ___ ___ ___ tortillas**<br>**¿Cuántas tortillas se hacen al día? ___ ___ ___ ___ tortillas**		

III.26 ¿Qué clase de combustible usa usted/***Usan ustedes*** para preparar la mase y hacer las tortillas?

**Opciones de respuesta**	**Códigos**	**Opciones de respuesta**	**Códigos**
**1. Leña**		**4. Electricidad**	
**2. Carbón**		**5. Otro (E)**	
**3. Gas propano**			

III.27 ¿De dónde consiguen el agua que usan para hacer la masa de las tortillas?

**Opciones de respuesta**	**Códigos**	**Opciones de respuesta**	**Códigos**
**1. Chorro propio**		**4. Pozo público/de pariente o vecino**	
**2. Chorro público /de pariente o vecino**		**5. Río, lago, manantial**	
**3. Pozo propio**		**Otro (E)**	

**III.28** ¿Qué hace usted/***hacen ustedes*** con el agua que usan para el nixtamal y el lavado?<br>---<br>***¿Qué harían usted/ustedes con….?***	1. La usan de nuevo para remojar, nixtamalizar o desaguar<br>2. La usan para preparar otros alimentos<br>3. La dan a los animales ***(cerdos, aves de corral)***<br>4. La tiran o descartan<br>5. Otro ***(especifique)*** ___________________________________	1 ____<br>2 ____<br>3 ____<br>4 ____<br>5 ____
	6. No sabe	
**III.29** Después de preparar las tortillas, ¿Qué hace usted/ ***hacen ustedes*** con la masa que le/s sobra?<br>---<br>***¿Qué haría usted/ que harían ustedes…?***	1 La guardan para hacer tortillas del siguiente tiempo/día<br>2. La usan para otras preparaciones<br>3. La descartan tiran o dan a los animales<br>4. Hace atol de masa /fresco de masa<br>5. Otro ***(especifique)*** ___________________________________	1 ____<br>2 ____<br>3 ____<br>4 ____<br>5 ____
	6. No sabe	6 ____

Cod Tortillería __ __ __

INSTRUMENTO DE RECOLECCIÓN DE DATOS -- Módulo 2: MUJER
"Estimando la exposición a micotoxinas y mejorando la inocuidad alimentaria en Guatemala"
(CIENSA, febrero de 2022)

Título del estudio: Estimación de exposición a micotoxinas e incremento de la seguridad alimentaria en Guatemala

Investigadores: Barbara Kowalcyk, Olga Torres, Ariel Garsow

Patrocinadores: Donante privado y The Ohio State University

Pegar calcomanía de ID AQUÍ

I. Información general de la mujer entrevistada

**0.** ID de la tortillería ____________________	
1. Departamento: ________________________________ 2. Código _____	**Guatemala = 1, Sololá = 2, Suchitepéquez = 3, Izabal = 4, Zacapa = 5.**
3. Municipio ____________________________________ 4. Código ___ ___	
5 Barrio/Colonia ____ 6. Código ___ ___	
7. Nombre de la tortillería __________________________ 8. Código______	9. Celular _________________
10. Roles de las personas que trabajan en la tortillería : 1. Dueña ___ 2. familiar ____ 3. empleada ____	
11. Persona **seleccionada** que da información de **Módulo Mujer**: 1. Dueña:___ 2. familiar ___ empleada ____	
12. Nombre de la persona entrevistada ____ 13. Edad ___ ___ años<br>***[Seleccione esta mujer entre quienes quiere participar en este estudio y dar una muestra de orina]***	
14. Idioma en la que se realizó la entrevista: **1. Español___ 2. Kaqchikel___ 3. Tz´utujil___ 4. K¨iche¨___ 5. Otro ___**<br>**15. Fecha ___ ___ /___ ___ / 2022 16. Encuestador/a ___________________________________ 17. Cod ____**	

II. Características de la mujer entrevistada y su hogar

Preguntas		Opciones de respuesta				Códigos
**II.1 Estado civil de la entrevistada**		***1. Casada 2. Unida 3. Soltera 4. Viuda 5. Divorciada 6. Separada***				
**II.2 Educación formal de la mujer entrevistada**	***¿Sabe leer?*** ___<br>***¿Sabe escribir?*** ___	_________________<br>***Grado***	____<br>***Código***	_________________<br>***Nivel***		____<br>***Código***
**II.3** ¿Dé donde es usted?, Dónde nació?	Comunidad ________________________________<br>Municipio _________________________________<br>Departamento ______________________________					Código ___ ___ ___<br>Código ___ ___ ___<br>Código ___ ___
**II.4** ¿Hace cuánto tiempo salió usted de su comunidad?	___ ___ **meses** ___ ___ **años**					

Parte III. Consumo de alimentos preparados a base de maíz, durante la última semana

No	Alimentos:	1 = Sí<br>0 = No	¿Cuántos días a la semana comió / Tomó?	¿Cuánto ____ comió /tomó en estos ____días?	Unidades	Gramos
	**Comió Usted ____ / Tomó usted ____ la semana pasada?**				*Nota: Considerar todos los alimentos consumidos por la persona objetivo, tanto en casa como fuera de ésta, independientemente donde se prepararon.*	
		Si lo comió o tomo…				
1	Tortillas	1 0	____	___ ___ ___. ___	u	40
2	Tamales	1 0	____	___ ___ ___. ___	u	200
3	Tamalitos	1 0	____	___ ___ ___ . ___	u	55
4	Chuchitos	1 0	____	___ ___ ___. ___	u	75
5	Shepes	1 0	____	___ ___ ___. ___	u	65
6	Tostadas	1 0	____	___ ___ ___. ___	u	25
7	Nachos	1 0	____	___ ___ ___. ___	u / b	5 / 60
8	Tortrix	1 0	____	___ ___ ___. ___	b	40
9	Pinol	1 0	____	___ ___ ___. ___	cd / oz / lb / tz	8 /28.7 / 454 / 95
10	Tayuyos / Tones	1 0	____	___ ___ ___. ___	u	55
11	Agua de masa	1 0	____	___ ___ ___. ___	v / tz / C	20 / 20/ 20
12	Atol de masa	1 0	____	___ ___ ___. ___	v / tz / C	40 / 40 / 40
13	Café de maíz	1 0	____	___ ___ ___. ___	cd / v / tz	12 /12 / 12
14	Atol de elote	1 0	____	___ ___ ___. ___	v / tz / C	40 / 40 / 40
15	Elote *(asado/cocido)*	1 0	____	___ ___ ___. ___	u	115
16	Maicena	1 0	____	___ ___ ___. ___	cd / oz / lb / tz	12 /28.7 /454 / 12
17	Polenta (CSB)	1 0	____	___ ___ ___. ___	cd / oz / lb / tz	14 /28.7 /454 / 14
18	Corn flakes	1 0	____	___ ___ ___. ___	cd / tz	7 / 45
19	Incaparina	1 0	____	___ ___ ___. ___	cd / oz / lb-b / bp tz	14 /28.7 /454 / 75 14
20	Tacos	1 0	____	___ ___ ___. ___	u	35
21	Otro alimento	1 0	____	___ ___ ___. ___	u / cd /oz /lb / _____	______

Abreviaturas de las unidades					
cd	=	cucharada	b	=	bolsa
C	=	cucharón	bp	=	bolsita *(bolsa pequeña)*
u	=	unidad	tz	=	taza
oz	=	onza	v	=	vaso

**lb**	**= libra**		
